# Supplementary material for: Molecular Structure, Vibrational Spectrum and Conformational Properties of 4-(4-Tritylphenoxy)phthalonitrile-Precursor for Synthesis of Phthalocyanines with Bulky Substituent
Source: Int J Mol Sci. 2022 Nov 11;23(22):13922. doi: 10.3390/ijms232213922 (PMC9696030; doi:10.3390/ijms232213922)
Supplement: Supplementary file 1 [file ijms-23-13922-s001.zip › ijms-1990635-supplementary.pdf]

SUPPLEMENTARY MATERIALS

Molecular structure, vibrational spectrum and conformational properties of 4-(4-tritylphenoxy)phthalonitrile - precursor for synthesis of phthalocyanines with bulky substituent

Natalia V. Tverdova [1], Nina I. Giricheva [2], Vladimir E. Maizlish [3], Nikolay E. Galanin [3], Georgiy V. Girichev [1,*]

[1] Department of Physics, Ivanovo State University of Chemistry and Technology, Sheremetevsky Avenue 7, 153000 Ivanovo Russia

[2] Nanomaterial Research Institute, Ivanovo State University, Ermak street 39, 153025 Ivanovo, Russia

[3] Department of Fine Organic Chemistry and Technology, Ivanovo State University of Chemistry and Technology, Sheremetevsky Avenue 7, 153000 Ivanovo Russia

* Correspondence: g.v.girichev@mail.ru; girichev@isuct.ru

Content

1. **Section S1.** Synthesis and characterization of 4-(4-tritylphenoxy)phthalonitrile sample …...2
2. **Table S1.** Selected characteristics of 4-(4-tritylphenoxy)phthalonitrile molecule according to DFT calculations……………………………….……………….……….…..........6
3. **Table S2.** Relative Gibbs energies (ΔG^0_{478}, kcal/mol), and molar fractions χ_i of conformers (mol%) in saturated vapor calculated for T=478K according to DFT calculations…..……………………………………………….……… ……….......7
4. **Table S3.** Theoretical (ω_{theor}) vibrations frequencies of conformers and experimental (ν_{exp}) of 4-(4-tritylphenoxy)phthalonitrile. Nonscaled theoretical frequencies are given……………………………………………………………………….……8
5. **Figure S1.** The calculated IR spectra of the conformers I and II (*cis*) and III and IV (*trans*)……………. ..……………………………………………….…………..19
6. **Figure S2.** Theoretical molecular intensities sM(s) of conformers I-IV of 4-(4-tritylphenoxy)phthalonitrile………………………………………………… 19
7. **Figure S3.** Theoretical radial distribution function f(r) for conformer II and difference Δf(r) for conformers I, III, IV…………..…………………………………………..…20
8. **Table S4.** Conditions of the synchronous GED/MS experiment…….…………..……….…..20

S1. Synthesis details

S1-1. Introduction

Among the huge number of phthalocyanines synthesized to date, a special place is occupied by compounds containing trityl groups in their composition. Such phthalocyanines include, among others, 2(3),9(10),16(17),23(24)-tetrakis-(4-tritylphenoxy)phthalocyanine and its complexes with various metals (**1a**–**1d**). *Scheme 1-1.*

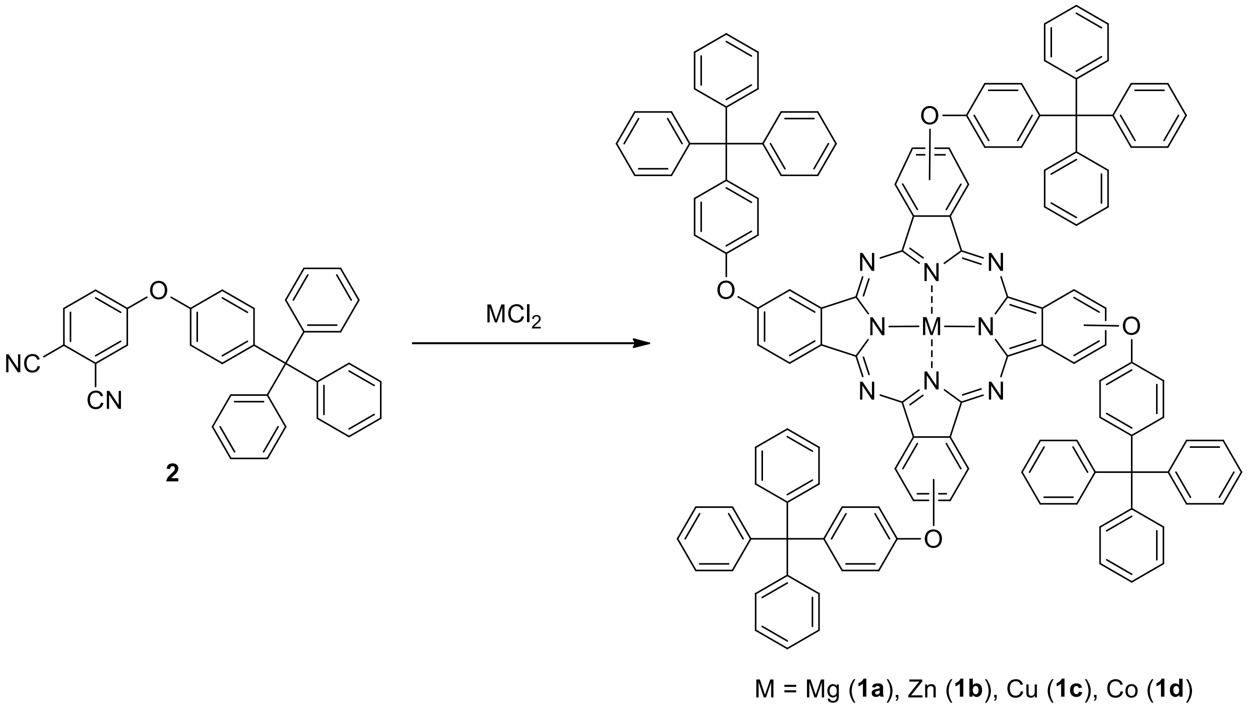

Scheme 1-1.

The presence of bulky trityl groups in phthalocyanine and its complexes determines their liquid-crystal properties [1–4]; in addition, these compounds are promising for use in thin-film electronics [5].

The starting compound for the synthesis of phthalocyanines of group (**1**) is 4-(4-tritylphenoxy)phthalonitrile **2**. Here we present the full cycle of its synthesis, consisting of the steps of benzene alkylation with carbon tetrachloride, phenol alkylation with triphenylmethanol, and nucleophilic substitution of the nitro group in 4-nitrophthalonitrile with the 4-tritylphenol. The scheme of chemical transformations is shown in *Scheme S1-2*.

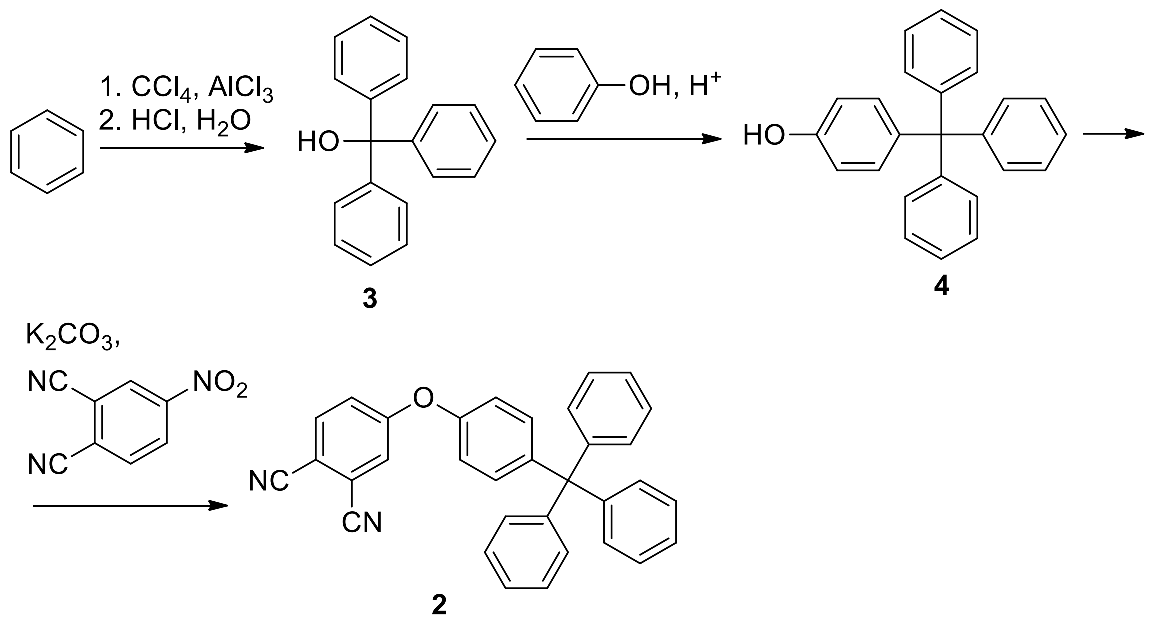

Scheme S1-2. Synthesis of 4-(4-tritylphenoxy)phthalonitrile **2**.

The first step in the synthesis is the alkylation of benzene. It is taken in a significant excess relative to carbon tetrachloride to maintain the mobility of the reaction mass. Alkylation yields triphenylchloromethane, which is then hydrolyzed to triphenylmethanol **3** by refluxing with hydrochloric acid. Its yield is about 60%. The unreacted benzene can be distilled off, dried, and reused in the synthesis. At the second stage, alcohol **3** is used for phenol alkylation. The reaction is carried out in an acetic acid medium catalyzed by sulfuric acid; as a result, 4-tritylphenol **4** is formed with a yield of 56%. At the third stage, phenol **4** reacts with 4-nitrophthalonitrile to form target 4-(4-tritylphenoxy)phthalonitrile **2** in 89% yield. The total yield of nitrile **2**, based on the starting carbon tetrachloride, was about 30%. The moderate yield is offset by the availability and low cost of reagents. The composition and structure of compounds **2** – **4** were confirmed by elemental analysis, mass spectra and ^{1}H NMR spectra. In addition, ^{13}C NMR spectrum.

S1-2. Experimental section

S1-2.1. Materials and measurements

Chloroform, tetrachloromethane and benzene of chemical grade by EKOS (http://www.ekos-1.ru) were used without further purification. 4-Nitrophthalonitrile were purchased from Aldrich (www.sigmaaldrich.com). The silica-gel 60 (0.063–0.200 mm) were from Merck (www.merck.de).

Mass spectroscopy measurements in both LDI-TOF (matrix-free) modes were performed with a Shimadzu AXIMA Confidence spectrometer. ^{1}H NMR (500.13 MHz) and ^{13}C NMR (125 MHz) spectra were recorded on a Bruker Avance-500 spectrometer in $CDCl_3$. Spectra were referenced internally by using the residual solvent resonance (δ = 7.28 (^{1}H) and 77.09 (^{13}C) ppm for $CDCl_3$) relative to $SiMe_4$ (TMS). Elemental analyses were performed on an FlashEA 1112 CHNS–O elemental analyzer. The infrared spectra were recorded for the films deposited from solution on the KRS-5 slides in the range of 400–4000 cm^{-1} using an Avatar 360 FT-IR spectrometer.

*S1-2.2. Synthesis of triphenylmethanol (**3**).*

To a mixture of 100.0 g (1.28 mol) of dry benzene and 20.0 g (0.13 mol) of dry carbon tetrachloride was added 20.0 g (0.15 mol) of anhydrous aluminum chloride at room temperature in portions of 5 g over an hour, heated to boiling and kept for another 6 h, after to which 100 ml of 10% hydrochloric acid was added and stirred at the boil for 1 h. The organic layer was separated, benzene and carbon tetrachloride that did not enter into the reaction were distilled off, the residue was recrystallized from propan-2-ol. Yield 20.0 g (58%). White substance, soluble in chloroform, benzene, DMSO. MS (LDI–TOF, negative mode): m/z 259.35 [Calc. for $[M–H]^{-}$: 259.12]. Another peak: m/z 243.35 $[M–OH]^{-}$. ^{1}H NMR ($CDCl_3$), ppm: 7.43–7.33 (m, 15H, Ar–H), 3.01 s (1H, O–H). Anal. Calc. (%) for $C_{19}H_{16}O$: C 87.66, H 6.19. found: C 87.22, H 6.33.

*S1-2.3. Synthesis of 4-tritylphenol (**4**).*

A mixture of 16.0 g (0.06 mol) of compound 3 and 7.0 g (0.07 mol) of phenol was dissolved in 30 ml of acetic acid, 3 ml of concentrated sulfuric acid was added, and refluxed for 10 h. 10% sodium hydroxide solution, 200 ml water, 20 ml acetone, then recrystallized from propan-2-ol. Yield 11.6 g (56%). White substance, soluble in chloroform, benzene, DMSO. MS (LDI–TOF, negative mode): m/z 335.48 [Calc. for $[M–H]^{-}$: 335.15]. ^{1}H NMR ($CDCl_3$), ppm: 7.29–7.26 (m, 6H, Ar–H), 7.23–7.20 (m, 9H, Ar–H), 7.10 (d, 2H, J=8.9 Hz, Ar–H), 6.75 (d, 2H, J=8.8 Hz, Ar–H), 4.72 (br, 1H, O–H). Anal. Calc. (%) for $C_{25}H_{20}O$: C 89.25, H 5.99. found: C 89.21, H 6.04.

*S1-2.4. Synthesis of 4-(4-tritylphenoxy)phthalonitrile (**2**).*

A mixture of 2.6 g (0.015 mol) of 4-nitrophthalonitrile, 6.9 g (0.02 mol) of compound **4**, 3.0 g of K_2CO_3, and 50 ml of DMF was stirred for 10 h at 110°C, cooled, and poured into 300 ml of water. The precipitate was filtered off, washed with 200 ml of water, dried in air at 80°C, and recrystallized from propan-2-ol. Yield 6.2 g (89%), light yellow substance, soluble in chloroform, benzene, DMSO. Rf 0.59 (chloroform, Silufol). MS (LDI–TOF, negative mode): m/z 461.96 [Calc. for $[M]^-$: 462.17]. ^{1}H NMR ($CDCl_3$), ppm: 7.77 (d, 1H, J=8.7 Hz, Ar–H), 7.37–7.26 (m, 19H, Ar–H), 7.00 (d, 2H, J=8.6 Hz, Ar–H). ^{13}C NMR ($CDCl_3$), ppm: 161.56, 151.50, 146.35, 145.12, 135.46, 133.36, 131.04, 127.73, 126.26, 121.63, 119.35, 117.66, 115.05, 108.92, 64.67. FT–IR spectrum (ν, cm^{-1}): 3057(C–H), 2231(C≡N), 1593(C=C), 1566, 1491, 1311, 1249 (Ar–O–Ar), 1213, 1176, 753, 704, 632. Anal. Calc. (%) for $C_{33}H_{22}N_2O$: C 85.68, H 4.80, N 6.06. found: C 85.77, H 4.91, N 5.99.

References

1. Usol'tseva N., Bykova V., Ananjeva G., Zharnikova N., Kudrik E. Mesomorphism and Glass Formation of Phthalocyanine Metal Complexes with Bulky Substituents. Mol. Cryst. Liq. Cryst., 2004. Vol. 411, pp. 329–336 (https://doi.org/10.108015421400490435350).
2. Zharnikova N., Usol'tseva N., Kudrik E., Thelakkat M. Synthesis, mesomorphism and electrochemical properties of tetrasubstituted zinc and copper phthalocyanines. J. Mater. Chem., 2009, 19, 3161–3167 (do https://doi.org/10.1039/b821306b).
3. Nakamura H., Sugiyama K., Ohta K., Yasutake M. Phthalocyanine-based discotic liquid crystals switching from a molten alkyl chain type to a flying-seed-like type. *J. Mater. Chem. C,* 2017, 5, 7297–7306 (https://doi.org/10.1039/c7tc01197k).
4. Takagi Y., Ohta K., Shimosugi S., Fujii T., Itoh E. Flying-seed-like liquid crystals 2: unprecedented guidelines to obtain liquid crystalline compounds. *J. Mater. Chem.,* **2012**, *22, 14418-14425*. https://doi.org/10.1039/c2jm32284f.
5. Solgun D.G., Horoz S., Agirtas M.S. Synthesis of novel tetra(4-tritylphenoxy)substituted metallophthalocyanines and investigation of their aggregation, photovoltaic, solar cell properties. *Inorganic and Nano-Metal Chemistry*. 2018. 48:10, 508-514 (https://doi.org/10.1080/24701556.2019.1572624)

Table S1. Selected characteristics of 4-(4-tritylphenoxy)phthalonitrile molecule according to DFT calculations[1]

	B97D/cc-pVTZ				CAM-B3LYP/cc-pVTZ				PBE/cc-pVTZ			
	conformer				*conformer*				*conformer*			
	I	II	III	IV	I	II	III	IV	I	II	III	IV
ΔE_{total}, kcal/mol	0.428	0	0.828	0.496	0.122	0	0.345	0.266	0.129	0	0.371	0.308
E_{HOMO}, eV	-5.82	-5.84	-5.90	-5.90	-8.16	-8.16	-8.26	-8.23	-6.99	-6.99	-7.09	-7.06
E_{LUMO}, eV	-2.95	-2.91	-2.92	-2.88	-1.13	-1.11	-1.04	-1.03	-2.21	-2.20	-2.13	-2.13
μ, D	8.18	7.72	9.92	10.02	8.04	7.97	9.98	9.98	8.09	8.04	10.05	10.04
Parameters of a distorted tetrahedron CC_4 built from carbon atoms of four phenyl fragments												
C-C[2]	1.548	1.548	1.548	1.549	1.546	1.546	1.546	1.546	1.542	1.542	1.542	1.542
C-C-C[3]	113.3	113.4	113.3	113.3	111.4	111.5	111.3	111.5	111.3	111.5	111.2	111.4
C-C-C[4]	102.0	102.0	102.0	102.0	105.6	105.4	105.9	105.5	106.0	105.6	106.0	105.7
Parameters of Ph-O-PhN moiety												
C_{Ph}-O	1.403	1.404	1.400	1.401	1.389	1.389	1.388	1.388	1.386	1.386	1.385	1.385
O-C_{PhN}	1.364	1.365	1.364	1.365	1.352	1.352	1.352	1.353	1.350	1.350	1.350	1.350
C-C[5]	1.397	1.396	1.397	1.397	1.385	1.385	1.385	1.385	1.387	1.387	1.388	1.387
C-C[6]	1.403	1.404	1.403	1.403	1.388	1.388	1.388	1.388	1.392	1.392	1.391	1.391
C_8-C_9[2]	1.428	1.428	1.428	1.428	1.429	1.429	1.429	1.429	1.425	1.425	1.425	1.425
C≡N	1.163	1.163	1.163	1.163	1.145	1.145	1.145	1.145	1.152	1.152	1.152	1.152
C_{PhN}-O-C_{Ph}	118.7	117.6	119.8	118.8	119.9	119.8	120.0	120.0	119.2	119.2	119.5	119.4
C-C-C[5]	120.0	120.0	120.0	120.0	120.0	120.0	120.0	120.0	120.0	120.0	120.0	120.0
C-C-C[6]	120.0	120.0	120.0	120.0	120.0	120.0	120.0	120.0	120.0	120.0	120.0	120.0
Parameters of trityl group												
C-C[7]	1.398	1.399	1.398	1.398	1.386	1.386	1.386	1.386	1.389	1.389	1.389	1.389
C-C[7]	1.399	1.399	1.399	1.399	1.386	1.386	1.386	1.386	1.389	1.389	1.389	1.389
C-C[7]	1.399	1.399	1.399	1.399	1.386	1.386	1.386	1.386	1.389	1.389	1.389	1.389
C-C-C[7]	120.0	120.0	120.0	120.0	120.0	120.0	120.0	120.0	120.0	120.0	120.0	120.0
C-C-C[7]	120.0	120.0	120.0	120.0	120.0	120.0	120.0	120.0	120.0	120.0	120.0	120.0
C-C-C[7]	120.0	120.0	120.0	120.0	120.0	120.0	120.0	120.0	120.0	120.0	120.0	120.0

[1] distances in Å, bond angles in deg.; see Fig. 1 for numbering atoms, [2] averaged, [3] averaged value of four angles, [4] averaged value of two angles, [5] the average value in the PhO fragment, [6] the average value in the PhN fragment, [7] the average value in the phenyl ring

Table S2. Relative Gibbs energies (ΔG^0_{478}, kcal/mol), and molar fractions χ_i of conformers (mol%) in saturated vapor calculated for T=478K according to DFT calculations[1]

	I-IV forms	ΔG^0_{478}	χ_i		I-IV forms	ΔG^0_{478}	χ_i
B3LYP	I	0.28	29.1	CAM-B3LYP	I		27.3
	II	0.76	17.6		II	0.65	18.9
	III	0.95	14.3		III	0.80	16.2
	IV	0.0	39.0		IV	0.0	37.6
B97D	I	0.0	39.5	PBE	I	0.69	21.6
	II	0.74	18.1		II	0.85	18.4
	III	1.46	8.5		III	1.04	15.0
	IV	0.15	33.9		IV	0.0	45.0

[1] basis set cc-pVTZ

Table S3. Theoretical (ω_{theor}) vibrations frequencies of conformers and experimental (ν_{exp}) of 4-(4-tritylphenoxy)phthalonitrile. No scaled theoretical frequencies are given.

Sym-metry	ω_{theor}, cm^{-1}				ν_{exp}, cm^{-1}	Normal vibrational energy distribution in internal coordinates for *cis*-conformer II
	I	II	III	IV		
A	3224	3224	3217	3218		$\nu(C_{PhN}\text{-}H)$
A	3218	3218	3217	3217		$\nu(C_{Ph}\text{-}H)$
A	3215	3214	3215	3214	3083.84	$\nu(C_{thr}\text{ -}H)$
A	3215	3214	3214	3214		$\nu(C_{thr}\text{-}H)$
A	3212	3211	3213	3213		$\nu(C_{thr}\text{-}H)$
A	3210	3210	3213	3211		$\nu(C_{PhN}\text{-}H)$
A	3198	3198	3199	3199		$\nu(C_{Ph}\text{-}H)$
A	3197	3197	3197	3198		$\nu(C_{PhN}\text{-}H)$
A	3192	3193	3193	3192		$\nu(C_{thr}\text{-}H)$
A	3192	3192	3192	3192		$\nu(C_{thr}\text{-}H)$
A	3192	3191	3191	3191		$\nu(C_{thr}\text{-}H)$, $\nu(C_{Ph}\text{-}H)$
A	3190	3190	3190	3190		$\nu(C_{Ph}\text{-}H)_{Ph}$, $\nu(C_{thr}\text{-}H)$
A	3185	3185	3185	3184	3056.83	$\nu(C_{thr}\text{-}H)$, $\nu(C_{Ph}\text{-}H)$
A	3184	3183	3183	3183		$\nu(C\text{-}H)_{Ph}$, $\nu(C_{thr}\text{-}H)$
A	3183	3183	3183	3183		$\nu(C_{thr}\text{-}H)$, $\nu(C_{Ph}\text{-}H)$
A	3183	3183	3182	3181		$\nu(C_{thr}\text{-}H)$, $\nu(C_{Ph}\text{-}H)_{Ph}$
A	3174	3173	3173	3173		$\nu(C_{thr}\text{-}H)$
A	3172	3172	3172	3172		$\nu(C_{thr}\text{-}H)$
A	3172	3172	3172	3171		$\nu(C_{thr}\text{-}H)$
A	3165	3165	3164	3164		$\nu(C_{thr}\text{-}H)$
A	3164	3164	3164	3164		$\nu(C_{thr}\text{-}H)$
A	3164	3164	3163	3163		$\nu(C_{thr}\text{-}H)$
A	2344	2344	2345	2345		$\nu(N\text{-}C_N)$, $\nu(C_{PhN}\text{-}C_N)$
A	2338	2338	2338	2338	2233.25	$\nu(N\text{-}C_N)$, $\nu(C_{PhN}\text{-}C_N)$
A	1649	1649	1651	1650		$\nu(C\text{-}C)_{ring\text{-}all}$, $\delta(CCC)_{ring\text{-}all}$, $\delta(CCH)_{ring\text{-}all}$
A	1644	1644	1645	1645		$\nu(C_{Ph}\text{-}C_{Ph})_{ring}$, $\nu(C_{thr}\text{-}C_{thr})_{ring}$, $\delta(CCC)_{ring\text{-}all}$, $\delta(C_{thr}C_{thr}H)$
A	1640	1641	1641	1641		$\nu(C_{thr}\text{-}C_{thr})_{ring}$, $\delta(C_{thr}C_{thr}C_{thr})_{ring}$, $\delta(C_{thr}C_{thr}H)$
A	1640	1640	1640	1640		$\nu(C_{thr}\text{-}C_{thr})_{ring}$, $\delta(C_{thr}C_{thr}C_{thr})_{ring}$, $\delta(C_{thr}C_{thr}H)$
A	1631	1631	1633	1633	1592.9	$\nu(C_{PhN}\text{-}C_{PhN})_{ring}$, $\nu(C_{Ph}\text{-}C_{Ph})_{ring}$, $\delta(OC_{PhN}C_{PhN})$, $\delta(CCC)_{ring\text{-}all}$, $\delta(CCH)_{ring\text{-}all}$
A	1626	1626	1626	1626		$\nu(C_{thr}\text{-}C_{thr})_{ring}$, $\nu(C_{Ph}\text{-}C_{Ph})_{ring}$, $\delta(CCC)_{ring\text{-}all}$, $\delta(CCH)_{ring\text{-}all}$
A	1621	1621	1621	1621		$\nu(C_{thr}\text{-}C_{thr})_{ring}$, $\delta(CCC)_{C4}$, $\delta(C_{thr}C_{thr}C_{thr})_{ring}$, $\delta(C_{thr}C_{thr}H)$
A	1621	1621	1621	1621		$\nu(C_{Ph}\text{-}C_{Ph})_{ring}$, $\nu(C_{thr}\text{-}C_{thr})_{ring}$, $\delta(C_{thr}C_{thr}C_{thr})_{ring}$, $\delta(C_{thr}C_{thr}H)$, $\delta(C_{Ph}C_{Ph}C_{Ph})_{ring}$, $\delta(C_{Ph}C_{Ph}H)$
A	1620	1619	1620	1619		$\nu(C_{Ph}\text{-}C_{Ph})_{ring}$, $\nu(C_{thr}\text{-}C_{thr})_{ring}$, $\delta(C_{thr}C_{thr}C_{thr})_{ring}$, $\delta(C_{thr}C_{thr}H)$, $\delta(C_{Ph}C_{Ph}C_{Ph})_{ring}$, $\delta(C_{Ph}C_{Ph}H)$, $\delta(OC_{Ph}C_{Ph})$
A	1596	1596	1593	1593		$\nu(C_{PhN}\text{-}C_{PhN})_{ring}$, $\delta(C_{Ph}C_{Ph}C_{Ph})_{ring}$, $\delta(C_{Ph}C_{Ph}H)$,

						$\delta(OC_{PhN}C_{PhN})$, $\delta(C_{PhN}C_{PhN}C_{PhN})_{ring}$, $\delta(C_{PhN}C_{PhN}H)$, bending- $\rho(C_{PhN}C_{N}N)$
A	1539	1539	1540	1540		$\nu(C_{Ph}$-$C_{Ph})_{ring}$, $\nu(O$-$C_{Ph})$, $\delta(CCH)_{ring\text{-}all}$, $\delta(C_{Ph}C_{Ph}C_{Ph})_{ring}$, $\delta(C_{Ph}C_{Ph}H)$, $\delta(OC_{PhN}C_{PhN})$, $\delta(C_{PhN}C_{PhN}C_{PhN})_{ring}$, $\delta(C_{PhN}C_{PhN}H)_{ring}$
A	1533	1533	1533	1533		$\nu(C_{thr}$-$C_{thr})_{ring}$, $\delta(C_{thr}C_{thr}H)$
A	1532	1532	1532	1531		$\nu(C_{thr}$-$C_{thr})_{ring}$, $\delta(C_{thr}C_{thr}C_{thr})_{ring}$, $\delta(C_{thr}C_{thr}H)$
A	1531	1531	1531	1531		$\nu(C_{thr}$-$C_{thr})_{ring}$, $\delta(C_{thr}C_{thr}C_{thr})_{ring}$, $\delta(C_{thr}C_{thr}H)$
A	1526	1526	1522	1522	1490.68	$\nu(C_{PhN}$-$C_{PhN})_{ring}$, $\delta(C_{PhN}C_{PhN}H)_{ring}$, $\nu(C_{PhN}$-$O)$, $\nu(C_{PhN}$-$C_{N})$,
A	1484	1484	1485	1484		$\nu(C_{thr}$-$C_{thr})_{ring}$, $\delta(C_{thr}CC_{thr})$, $\delta(CC_{thr}C_{thr})$, $\delta(C_{thr}C_{thr}C_{thr})_{ring}$, $\delta(C_{thr}C_{thr}H)$
A	1480	1481	1481	1480		$\nu(C_{thr}$-$C_{thr})_{ring}$, $\delta(C_{thr}CC_{thr})$, $\delta(CC_{thr}C_{thr})$, $\delta(C_{thr}C_{thr}C_{thr})_{ring}$, $\delta(C_{thr}C_{thr}H)$
A	1477	1477	1477	1477		$\nu(C_{thr}$-$C_{thr})_{ring}$, $\delta(C_{thr}CC_{thr})$, $\delta(CC_{thr}C_{thr})$, $\delta(C_{thr}C_{thr}C_{thr})_{ring}$, $\delta(C_{thr}C_{thr}H)$
A	1444	1444	1451	1451		$\nu(C_{PhN}$-$O)$, $\nu(C_{PhN}$-$C_{PhN})_{ring}$, $\nu(C_{PhN}$-$C_{N})$, $\delta(OC_{PhN}C_{PhN})$, $\delta(C_{PhN}C_{PhN}C_{PhN})_{ring}$, $\delta(C_{PhN}C_{PhN}H)$, $\delta(C_{PhN}C_{PhN}C_{N})$
A	1438	1438	1438	1438		$\nu(C_{Ph}$-$C_{Ph})_{ring}$, $\delta(C_{Ph}C_{Ph}C_{Ph})_{ring}$, $\delta(OC_{Ph}C_{Ph})$, $\delta(C_{Ph}C_{Ph}H)$
A	1372	1372	1371	1371		$\delta(C_{thr}C_{thr}H)$, $\nu(C_{thr}$-$C_{thr})_{ring}$, $\delta(CC_{thr}C_{thr})$,
A	1370	1370	1369	1370		$\nu(C_{thr}$-$C_{thr})_{ring}$, $\delta(CC_{thr}C_{thr})$, $\delta(C_{thr}C_{thr}H)$
A	1366	1367	1366	1366		$\nu(C_{thr}$-$C_{thr})_{ring}$, $\delta(C_{thr}C_{thr}H)$
A	1342	1340	1342	1341		$\nu(C_{PhN}$-$O)$, $\nu(C_{PhN}$-$H)$, $\delta(C_{PhN}C_{PhN}H)$, $\delta(C_{Ph}C_{Ph}H)$
A	1340	1340	1328	1328	1311.3	$\nu(C_{PhN}$-$O)$, $\nu(C_{Ph}$-$O)$, $\nu(C_{PhN}$-$C_{PhN})_{ring}$, $\delta(C_{PhN}C_{PhN}C_{PhN})_{ring}$, $\delta(C_{PhN}C_{PhN}H)$
A	1328	1328	1325	1325		$\nu(C_{thr}$-$C_{thr})_{ring}$, $\nu(C_{Ph}$-$C_{Ph})_{ring}$, $\delta(CC_{thr}C_{thr})$, $\delta(C_{thr}CC_{thr})$, $\delta(C_{thr}C_{thr}H)$
A	1318	1319	1318	1318		$\nu(C_{thr}$-$C_{thr})_{ring}$, $\delta(C_{Ph}CC_{thr})$, $\delta(CC_{thr}C_{thr})$, $\delta(C_{thr}C_{thr}H)$
A	1317	1317	1317	1316		$\nu(C_{thr}$-$C_{thr})_{ring}$, $\delta(C_{Ph}CC_{thr})$, $\delta(CC_{thr}C_{thr})$, $\delta(C_{thr}C_{thr}H)$
A	1314	1314	1313	1314		$\nu(C_{Ph}$-$C_{Ph})_{ring}$, $\nu(C_{thr}$-$C_{thr})_{ring}$, $\delta(C_{Ph}C_{Ph}C)$, $\delta(C_{thr}C_{thr}H)$, $\delta(C_{Ph}CC_{thr})$, $\delta(OC_{Ph}C_{Ph})$, $\tau(C_{Ph}CC_{thr}C_{thr})$
A	1308	1308	1312	1312	1282.37	$\nu(C_{PhN}$-$O)$, $\nu(C_{Ph}$-$O)$, $\nu(C_{PhN}$ -$C_{PhN})_{ring}$, $\delta(C_{PhN}C_{PhN}H)$, $\delta(OC_{PhN}C_{PhN})$, $\delta(C_{PhN}OC_{Ph})$, $\delta(C_{PhN}C_{PhN}C_{PhN})_{ring}$
A	1272	1271	1276	1276	1249.58	$\nu(C_{PhN}$-$O)$, $\nu(C_{PhN}$-$C_{PhN})_{ring}$, $\nu(C_{PhN}$-$C_{N})$, $\delta(OC_{PhN}C_{PhN})$, $\delta(C_{PhN}C_{PhN}C_{PhN})_{ring}$, $\delta(C_{PhN}C_{PhN}H)$
A	1236	1235	1239	1238	1212.94	$\nu(C_{Ph}$-$O)$, $\nu(C_{Ph}$-$C_{Ph})_{ring}$, $\nu(C_{PhN}$ -$C_{PhN})_{ring}$, $\delta(C_{Ph}C_{Ph}C_{Ph})_{ring}$, $\delta(C_{Ph}C_{Ph}H)$, $\delta(C_{thr}C_{thr}H)$
A	1227	1228	1225	1225		$\nu(C_{PhN}$-$C_{PhN})_{ring}$, $\nu(C_{PhN}$-$O)$, $\delta(OC_{PhN}C_{PhN})$,

						$\delta(C_{PhN}C_{PhN}C_{PhN})_{ring}$, $\delta(C_{PhN}C_{PhN}H)$
A	1221	1222	1221	1221		$\nu(C_{thr}\text{-}C_{thr})_{ring}$, $\delta(C_{thr}C_{thr}H)$
A	1219	1220	1219	1219		$\nu(C_{thr}\text{-}C_{thr})_{ring}$, $\delta(C_{thr}C_{thr}H)$
A	1219	1219	1218	1218		$\nu(C_{thr}\text{-}C_{thr})_{ring}$, $\delta(C_{thr}C_{thr}H)$
A	1199	1199	1202	1201	1176.29	$\nu(C_{Ph}\text{-}O)$, $\nu(C\text{-}C)_{ring\text{-}all}$, $\delta(C_{Ph}C_{Ph}H)$, $\delta(C_{PhN}C_{PhN}H)$
A	1195	1196	1192	1195		$\nu(C\text{-}C_{thr})$, $\nu(C_{Ph}\text{-}C_{Ph})_{ring}$, $\delta(C_{Ph}CC_{thr})$, $\delta(C_{Ph}C_{Ph}H)$, $\delta(CC_{thr}C_{thr})$, $\tau(CC_{thr}C_{thr}C_{thr})$, $\tau(C_{Ph}CC_{thr}C_{thr})$, $\delta(C_{thr}C_{thr}H)$
A	1188	1188	1187	1188		$\nu(C_{thr}\text{-}C_{thr})_{ring}$, $\delta(C_{thr}C_{thr}H)$
A	1187	1187	1187	1187		$\nu(C_{thr}\text{-}C_{thr})_{ring}$, $\delta(C_{thr}C_{thr}H)$
A	1187	1186	1187	1187		$\nu(C_{thr}\text{-}C_{thr})_{ring}$, $\delta(C_{thr}C_{thr}H)_{ring}$
A	1184	1184	1183	1183		$\nu(C_{Ph}\text{-}O)$, $\nu(C_{PhN}\text{-}O)$, $\nu(C_{PhN}\text{-}C)_{ring}$, $\delta(C_{PhN}C_{PhN}C_{PhN})_{ring}$, $\delta(C_{PhN}C_{PhN}H)_{ring}$, $\delta(C_{Ph}C_{Ph}C_{Ph})_{ring}$, $\delta(C_{Ph}C_{Ph}H)$
A	1172	1172	1172	1172		$\nu(C_{thr}\text{-}C_{thr})_{ring}$, $\delta(CC_{Ph}C_{Ph})$, $\delta(C_{thr}CC_{thr})$, $\delta(CC_{thr}C_{thr})$, $\delta(C_{thr}C_{thr}C_{thr})$, $\delta(C_{thr}C_{thr}H)$, $\tau(C_{Ph}CC_{thr}C_{thr})$
A	1170	1171	1171	1170		$\nu(C_{Ph}\text{-}C)$, $\nu(C_{thr}\text{-}C)$, $\nu(C_{thr}\text{-}C_{thr})_{ring}$, $\delta(C_{Ph}CC_{thr})$, $\delta(C_{Ph}C_{Ph}C_{Ph})_{ring}$, $\delta(C_{Ph}C_{Ph}H)$, $\delta(CC_{thr}C_{thr})$, $\delta(C_{thr}C_{thr}C_{thr})_{ring}$, $\delta(C_{thr}C_{thr}H)$, $\tau(C_{Ph}CC_{thr}C_{thr})$, $\tau(CC_{thr}C_{thr}C_{thr})$
A	1159	1159	1159	1159		$\nu(C_{thr}\text{-}C)$, $\nu(C_{Ph}\text{-}C_{Ph})_{ring}$, $\nu(C_{thr}\text{-}C_{thr})_{ring}$, $\delta(C_{Ph}C_{Ph}C_{Ph})_{ring}$, $\delta(C_{Ph}C_{Ph}H)$, $\delta(CC_{Ph}C_{Ph})$, $\delta(CC_{thr}C_{thr})$, $\delta(C_{thr}C_{thr}H)$
A	1137	1137	1138	1137		$\nu(C_{Ph}\text{-}C_{Ph})_{ring}$, $\delta(C_{Ph}C_{Ph}C_{Ph})_{ring}$, $\delta(C_{Ph}C_{Ph}H)$
A	1115	1115	1116	1116		$\nu(C_{thr}\text{-}C_{thr})_{ring}$, $\delta(CC_{thr}C_{thr})$, $\delta(C_{thr}C_{thr}H)$
A	1114	1114	1115	1115		$\nu(C_{thr}\text{-}C_{thr})_{ring}$, $\delta(CC_{thr}C_{thr})$, $\delta(C_{thr}C_{thr}H)$
A	1111	1112	1113	1114		$\nu(C_{thr}\text{-}C_{thr})_{ring}$, $\delta(CC_{thr}C_{thr})$, $\delta(C_{thr}C_{thr}H)$
A	1111	1111	1112	1111	1085.64	$\nu(C_{PhN}\text{-}C_{N})$, $\delta(C_{PhN}C_{PhN}H)$, $\nu(C_{PhN}\text{-}O)$, $\nu(C_{PhN}\text{-}C_{PhN})_{ring}$, $\delta(C_{PhN}C_{PhN}C_{PhN})_{ring}$, $\delta(C_{PhN}C_{PhN}C_{N})$ $\delta(C_{thr}C_{thr}H)$
A	1060	1060	1060	1060		$\nu(C_{thr}\text{-}C_{thr})_{ring}$, $\nu(C_{thr}\text{-}C)$, $\delta(C_{thr}C_{thr}H)_{ring}$, $\tau(CC_{thr}C_{thr}C_{thr})$
A	1059	1059	1059	1059		$\nu(C_{thr}\text{-}C)$, $\nu(C_{thr}\text{-}C_{thr})_{ring}$, $\delta(C_{thr}C_{thr}C_{thr})_{ring}$, $\delta(C_{thr}C_{thr}H)$
A	1057	1057	1058	1058		$\nu(C_{thr}\text{-}C_{thr})_{ring}$, $\delta(C_{thr}C_{thr}C_{thr})_{ring}$, $\delta(C_{thr}C_{thr}H)$
A	1040	1039	1041	1040		$\nu(C_{Ph}\text{-}C_{Ph})_{ring}$, $\nu(C_{Ph}\text{-}O)$, $\delta(C_{Ph}C_{Ph}C_{Ph})_{ring}$, $\delta(C_{Ph}C_{Ph}H)$, $\delta(CC_{Ph}C_{Ph})$, $\delta(OC_{Ph}C_{Ph})$
A	1023	1023	1024	1023		$\nu(C_{thr}\text{-}C_{thr})_{ring}$, $\delta(CC_{thr}C_{thr})$, $\delta(C_{thr}C_{thr}C_{thr})_{ring}$, $\delta(C_{thr}C_{thr}H)$
A	1023	1023	1023	1023		$\nu(C_{thr}\text{-}C_{thr})_{ring}$, $\delta(CC_{thr}C_{thr})$, $\delta(C_{thr}C_{thr}C_{thr})_{ring}$, $\delta(C_{thr}C_{thr}H)_{ring}$
A	1023	1023	1023	1023		$\nu(C_{thr}\text{-}C_{thr})_{ring}$, $\delta(CC_{thr}C_{thr})$, $\delta(C_{thr}C_{thr}C_{thr})_{ring}$, $\delta(C_{thr}C_{thr}H)_{ring}$
A	1014	1017	1014	1014		waving- $\sigma(C_{thr}\text{ -}H)$, $\tau(C_{Ph}CC_{thr}C_{thr})$, $\tau(CC_{thr}C_{thr}C_{thr})$, $\tau(C_{thr}C_{thr}C_{thr}C_{thr})_{ring}$

A	1013	1013	1013	1013		waving- $\sigma(C_{Ph}\text{-}H)$, waving- $\sigma(C_{thr}\text{ -}H)$, $\tau(CC_{thr}C_{thr}C_{thr})$, $\tau(C_{thr}C_{thr}C_{thr}C_{thr})_{ring}$
A	1012	1012	1012	1012		waving- $\sigma(C_{thr}\text{ -}H)$, $\tau(CC_{thr}C_{thr}C_{thr})$, $\tau(C_{thr}C_{thr}C_{thr}C_{thr})_{ring}$
A	1004	1005	1004	1004		waving- $\sigma(C_{Ph}\text{-}H)$, waving- $\sigma(C_{thr}\text{-}H)$, $\tau(C_{Ph}C_{Ph}C_{Ph}C_{Ph})_{ring}$, $\tau(C_{thr}C_{thr}C_{thr}C_{thr})_{ring}$
A	1002	1002	1001	1001		waving- $\sigma(C_{Ph}\text{-}H)$, waving- $\sigma(C_{thr}\text{-}H)$, $\tau(C_{Ph}C_{Ph}C_{Ph}C_{Ph})_{ring}$, $\tau(C_{thr}C_{thr}C_{thr}C_{thr})_{ring}$
A	1000	1001	999	1000		waving- $\sigma(C_{Ph}\text{-}H)$, waving- $\sigma(C_{thr}\text{-}H)$, $\tau(C_{Ph}C_{Ph}C_{Ph}C_{Ph})_{ring}$, $\tau(C_{thr}C_{thr}C_{thr}C_{thr})_{ring}$
A	996	997	996	997		waving- $\sigma(C_{Ph}\text{-}H)$, waving- $\sigma(C_{thr}\text{-}H)$, $\tau(C_{Ph}C_{Ph}C_{Ph}C_{Ph})_{ring}$, $\tau(CC_{thr}C_{thr}C_{thr})$, $\tau(C_{thr}C_{thr}C_{thr}C_{thr})_{ring}$
A	994	994	991	991		waving- $\sigma(C_{Ph}\text{-}H)$, waving- $\sigma(C_{PhN}\text{-}H)$, waving- $\sigma(C_{PhN}\text{-}C_{N})$, $\tau(C_{PhN}C_{PhN}C_{PhN}C_{PhN})_{ring}$, $\tau(OC_{PhN}C_{PhN}C_{PhN})$
A	991	992	990	991		waving- $\sigma(C_{Ph}\text{-}H)$, waving- $\sigma(C_{Ph}\text{-}O)$, waving- $\sigma(C_{thr}\text{-}H)$, $\tau(C_{Ph}C_{Ph}C_{Ph}C_{Ph})_{ring}$, $\tau(C_{Ph}C_{Ph}OC_{PhN})$, $\tau(C_{thr}C_{thr}C_{thr}C_{thr})_{ring}$, $\tau(C_{Ph}CC_{thr}C_{thr})$
A	974	975	975	976		$\nu(C_{PhN}\text{-}O)$, $\nu(C_{PhN}\text{-}C_{PhN})_{ring}$, $\nu(C_{PhN}\text{-}C_{N})$, $\delta(CCC)_{C4}$, $\delta(C_{Ph}OC_{PhN})$, $\delta(OC_{PhN}C_{PhN})$, $\delta(C_{PhN}C_{PhN}C_{PhN})_{ring}$, $\delta(C_{PhN}C_{PhN}H)$, $\delta(C_{N}C_{PhN}C_{PhN})$, waving- $\sigma(C_{Ph}\text{-}H)_{Ph}$, waving- $\sigma(C_{Ph}\text{-}C)$, waving- $\sigma(C_{Ph}\text{-}O)$, waving- $\sigma(C_{thr}\text{-}C)$, waving- $\sigma(C_{thr}\text{-}H)$, $\tau(C_{Ph}C_{Ph}C_{Ph}C_{Ph})_{ring}$, $\tau(C_{Ph}CC_{thr}C_{thr})$
A	968	968	967	967		$\delta(CCC)_{C4}$, waving- $\sigma(C_{Ph}\text{-}H)$, waving- $\sigma(C\text{-}C_{thr})$, waving- $\sigma(C_{thr}\text{-}H)$, $\tau(C_{Ph}CC_{thr}C_{thr})$, $\tau(C_{thr}C_{thr}C_{thr}C_{thr})_{ring}$
A	960	960	960	959		$\delta(CCC)_{C4}$, waving- $\sigma(C\text{-}C_{thr})$, waving- $\sigma(C_{thr}\text{-}H)$, $\tau(C_{Ph}C_{Ph}CC_{thr})$, $\tau(C_{Ph}CC_{thr}C_{thr})$, $\tau(C_{thr}C_{thr}C_{thr}C_{thr})_{ring}$
A	956	958	957	956		$\delta(CCC)_{C4}$, waving- $\sigma(C_{Ph}\text{-}H)$, waving- $\sigma(C\text{-}C_{thr})$, waving- $\sigma(C_{thr}\text{-}H)$, $\tau(C_{Ph}CC_{thr}C_{thr})$, $\tau(C_{thr}C_{thr}C_{thr}C_{thr})_{ring}$
A	927	927	928	928		$\nu(C\text{-}C_{thr})_{C4}$, $\delta(CCC)_{C4}$, $\delta(CC_{thr}C_{thr})$, $\delta(C_{thr}C_{thr}C_{thr})_{ring}$, waving- $\sigma(C_{Ph}\text{-}H)$, waving- $\sigma(C_{Ph}\text{-}C)$, waving- $\sigma(C_{Ph}\text{-}O)$, waving- $\sigma(C_{thr}\text{-}H)$, $\tau(C_{Ph}C_{Ph}C_{Ph}C_{Ph})_{ring}$, $\tau(C_{Ph}CC_{thr}C_{thr})$, $\tau(CC_{thr}C_{thr}C_{thr})$
A	912	913	916	916		$\nu(C\text{-}C_{thr})_{C4}$, $\delta(CCC)_{C4}$, waving- $\sigma(C\text{-}C_{thr})$, waving- $\sigma(C_{thr}\text{-}H)$, waving- $\sigma(C_{PhN}\text{-}H)$, waving- $\sigma(C_{PhN}\text{ -}C_{N})$, $\tau(C_{Ph}OC_{PhN}C_{PhN})$, $\tau(C_{Ph}CC_{thr}C_{thr})$, $\tau(CC_{thr}C_{thr}C_{thr})$, $\tau(C_{PhN}C_{PhN}C_{PhN}C_{PhN})_{ring}$
A	912	913	912	912		$\delta(CCC)_{C4}$, waving- $\sigma(C\text{-}C_{thr})$, waving- $\sigma(C_{thr}\text{-}H)$, waving- $\sigma(O\text{-}C_{PhN})$, waving- $\sigma(C_{PhN}\text{-}H)$, waving- $\sigma(C_{PhN}\text{-}C_{N})$, $\tau(C_{Ph}C_{Ph}CC_{thr})$, $\tau(C_{Ph}OC_{PhN}C_{PhN})$, $\tau(C_{Ph}CC_{thr}C_{thr})$, $\tau(CC_{thr}C_{thr}C_{thr})$,

						$\tau(C_{PhN}C_{PhN}C_{PhN}C_{PhN})_{ring}$
A	910	912	911	911		$\nu(C-C_{thr})_{C4}$, $\delta(CCC)_{C4}$, $\delta(C_{thr}C_{thr}C_{thr})_{ring}$, waving- $\sigma(C-C_{thr})$, waving- $\sigma(C_{thr}-H)$, waving- $\sigma(C_{PhN}-H)$, waving- $\sigma(C_{PhN}-C_{N})$, $\tau(C_{Ph}C_{Ph}CC_{thr})$, $\tau(C_{Ph}OC_{PhN}C_{PhN})$, $\tau(C_{Ph}CC_{thr}C_{thr})$, $\tau(CC_{thr}C_{thr}C_{thr})$, $\tau(C_{PhN}C_{PhN}C_{PhN}C_{PhN})_{ring}$
A	874	874	874	873		waving- $\sigma(C_{Ph}-H)$, waving- $\sigma(C_{thr}-H)$, $\tau(C_{Ph}CC_{thr}C_{thr})$
A	872	873	872	872		$\delta(C_{Ph}OC_{PhN})$, $\delta(C_{Ph}C_{Ph}C_{Ph})_{ring}$, $\delta(CCC)_{C4}$, waving- $\sigma(C_{Ph}-H)$, waving- $\sigma(O-C_{Ph})$, waving- $\sigma(C_{thr}-H)$, $\tau(C_{Ph}C_{Ph}C_{Ph}C_{Ph})_{ring}$, $\tau(C_{Ph}C_{Ph}OC_{PhN})$, $\tau(C_{Ph}CC_{thr}C_{thr})$
A	868	869	867	867		$\delta(C_{Ph}OC_{PhN})$, waving- $\sigma(C_{Ph}-H)$, waving- $\sigma(O-C_{Ph})$, waving- $\sigma(C_{thr}-H)$, $\tau(C_{Ph}C_{Ph}C_{Ph}C_{Ph})_{ring}$, $\tau(C_{Ph}C_{Ph}OC_{PhN})$
A	867	867	866	866		waving- $\sigma(C_{Ph}-H)$, waving- $\sigma(C_{thr}-H)$
A	860	861	857	859	834.9	waving- $\sigma(C_{PhN}-C_{N})$, waving- $\sigma(C_{Ph}-H)$, waving- $\sigma(O-C_{PhN})$, waving- $\sigma(C_{PhN}-H)$, $\tau(C_{Ph}C_{Ph}OC_{PhN})$, $\tau(C_{Ph}OC_{PhN}C_{PhN})$, $\tau(OC_{PhN}C_{PhN}C_{PhN})$, $\tau(C_{PhN}C_{PhN}C_{PhN}C_{PhN})_{ring}$
A	852	853	849	849		$\nu(C_{Ph}-C_{Ph})_{ring}$, $\nu(O-C_{Ph})$, $\delta(C_{Ph}C_{Ph}C_{Ph})_{ring}$, $\delta(C_{Ph}C_{Ph}H)$, $\delta(CC_{Ph}C_{Ph})$, $\delta(OC_{PhN}C_{PhN})$, $\delta(C_{PhN}C_{PhN}C_{PhN})_{ring}$, $\delta(CCC)_{C4}$, waving- $\sigma(C_{Ph}-H)$, waving- $\sigma(C-C_{Ph})$, waving- $\sigma(O-C_{Ph})$, waving- $\sigma(C-C_{thr})$, waving- $\sigma(C_{thr}-H)$, $\tau(C_{Ph}C_{Ph}C_{Ph}C_{Ph})_{ring}$, $\tau(C_{Ph}CC_{thr}C_{thr})$, $\tau(C_{Ph}C_{Ph}OC_{PhN})$
A	848	849	840	841		$\delta(C_{Ph}C_{Ph}C_{Ph})_{ring}$, $\delta(OC_{PhN}C_{PhN})$, $\delta(C_{PhN}C_{PhN}C_{PhN})_{ring}$, waving- $\sigma(C_{Ph}-H)$, waving- $\sigma(C_{thr}-H)$, $\tau(C_{Ph}C_{Ph}C_{Ph}C_{Ph})_{ring}$
A	790	790	788	790		$\delta(CCC)_{C4}$, waving- $\sigma(C-C_{Ph})$, waving- $\sigma(C-C_{thr})$, waving- $\sigma(O-C_{Ph})$, waving- $\sigma(C_{thr}-H)$, $\tau(C_{Ph}C_{Ph}C_{Ph}C_{Ph})_{ring}$, $\tau(C_{Ph}C_{Ph}CC_{thr})$, $\tau(C_{Ph}CC_{thr}C_{thr})$, $\tau(C_{thr}C_{thr}C_{thr}C_{thr})_{ring}$
A	781	780	782	779		waving- $\sigma(C-C_{Ph})$, waving- $\sigma(O-C_{Ph})$, waving- $\sigma(C-C_{thr})$, waving- $\sigma(C_{thr}-H)$, $(C_{Ph}C_{Ph}C_{Ph}C_{Ph})_{ring}$, $\tau(C_{Ph}CC_{thr}C_{thr})$, $\tau(C_{thr}C_{thr}C_{thr}C_{thr})_{ring}$
A	769	769	769	768	751.96	waving- $\sigma(C_{thr}-H)$, waving- $\sigma(C-C_{thr})$, $\tau(C_{Ph}CC_{thr}C_{thr})$, $\tau(C_{thr}C_{thr}C_{thr}C_{thr})_{ring}$
A	764	766	765	767		$\delta(C_{Ph}OC_{PhN})$, waving- $\sigma(C_{Ph}-H)$, waving- $\sigma(C-C_{Ph})$, waving- $\sigma(O-C_{Ph})$, waving- $\sigma(C-C_{thr})$, waving- $\sigma(C_{thr}-H)$, $\tau(C_{Ph}C_{Ph}C_{Ph}C_{Ph})_{ring}$, $\tau(C_{Ph}C_{Ph}CC_{thr})$, $\tau(C_{Ph}C_{Ph}OC_{PhN})$, $\tau(C_{Ph}CC_{thr}C_{thr})$, $\tau(C_{thr}C_{thr}C_{thr}C_{thr})_{ring}$
A	756	756	754	753		waving- $\sigma(O-C_{PhN})$, waving- $\sigma(C_{PhN}-H)$, waving- $\sigma(C_{PhN}-C_{N})$, bending- $\rho(C_{PhN}C_{N}N)$, $\tau(C_{Ph}C_{Ph}OC_{PhN})$, $\tau(C_{Ph}OC_{PhN}C_{PhN})$, $\tau(OC_{PhN}C_{PhN}C_{PhN})$, $\tau(C_{PhN}C_{PhN}C_{PhN}C_{PhN})_{ring}$

A	744	744	737	737		$\nu(C_{PhN}{-}_{PhN}C)_{ring}$, $\nu(C_{PhN}{-}C_{N})$, $\delta(C_{Ph}OC_{PhN})$, $\delta(OC_{PhN}C_{PhN})$, $\delta(C_{PhN}C_{PhN}C_{PhN})_{ring}$, $\delta(C_{PhN}C_{PhN}H)$, $\delta(C_{PhN}C_{PhN}C_{N})_{ring}$, waving- $\sigma(C{-}C_{Ph})$, waving- $\sigma(C_{Ph}{-}H)$, waving- $\sigma(C_{thr}{-}H)$, $\tau(C_{Ph}C_{Ph}C_{Ph}C_{Ph})_{ring}$, $\tau(C_{Ph}C_{Ph}CC_{thr})$
A	728	730	727	727		waving- $\sigma(C{-}C_{thr})$, waving- $\sigma(C_{thr}{-}H)$, $\tau(C_{Ph}CC_{thr}C_{thr})$, $\tau(CC_{thr}C_{thr}C_{thr})$, $\tau(C_{thr}C_{thr}C_{thr}C_{thr})_{ring}$
A	725	725	724	724	703.74	waving- $\sigma(C_{thr}{-}H)$, $\delta(CCC)_{ringPhN}$, waving- $\sigma(C{-}C_{thr})$, $\tau(C_{Ph}C_{Ph}C_{Ph}C_{Ph})_{ring}$, $\tau(C_{Ph}CC_{thr}C_{thr})$, $\tau(C_{thr}C_{thr}C_{thr}C_{thr})_{ring}$
A	723	724	723	722		waving- $\sigma(C{-}C_{thr})$, waving- $\sigma(C_{thr}{-}H)$, $\tau(C_{Ph}CC_{thr}C_{thr})$, $\tau(CC_{thr}C_{thr}C_{thr})$, $\tau(C_{thr}C_{thr}C_{thr}C_{thr})_{ring}$
A	722	722	721	721		$\delta(C_{Ph}C_{Ph}C_{Ph})_{ring}$, $\delta(C_{thr}C_{thr}C_{thr})_{ring}$, $\delta(C_{PhN}C_{PhN}C_{PhN})_{ring}$, waving- $\sigma(C_{Ph}{-}H)$, waving- $\sigma(C{-}C_{thr})$, waving- $\sigma(C_{thr}{-}H)$,$\tau(C_{Ph}C_{Ph}C_{Ph}C_{Ph})_{ring}$, $\tau(C_{Ph}CC_{thr}C_{thr})$, $\tau(CC_{thr}C_{thr}C_{thr})$, $\tau(C_{thr}C_{thr}C_{thr}C_{thr})_{ring}$
A	696	697	694	695		$\delta(C_{Ph}OC_{PhN})$, $\delta(C_{thr}C_{thr}C_{thr})_{ring}$, $\delta(CC_{thr}C_{thr})$, $\delta(C_{thr}C_{thr}H)$, $\delta(C_{PhN}C_{PhN}C_{PhN})_{ring}$, waving- $\sigma(C_{Ph}{-}H)$, waving- $\sigma(C{-}C_{Ph})$, $\tau(C_{Ph}C_{Ph}C_{Ph}C_{Ph})_{ring}$, $\tau(C_{thr}C_{thr}C_{thr}C_{thr})_{ring}$
A	661	660	668	666		$\delta(C_{Ph}C_{Ph}C_{Ph})_{ring}$, $\delta(C_{Ph}C_{Ph}H)_{ring}$, $\delta(CC_{Ph}C_{Ph})$, $\delta(OC_{Ph}C_{Ph})$, $\delta(C_{thr}C_{thr}C_{thr})_{ring}$, waving- $\sigma(O{-}C_{PhN})$, $\tau(C_{Ph}OC_{PhN}C_{PhN})$, $\tau(C_{thr}C_{thr}C_{thr}C_{thr})_{ring}$, $\tau(OC_{PhN}C_{PhN}C_{PhN})$
A	659	658	660	660		$\delta(C_{Ph}C_{Ph}C_{Ph})_{ring}$, $\delta(C_{Ph}C_{Ph}H)$, $\delta(OC_{Ph}C_{Ph})$, waving- $\sigma(C{-}C_{Ph})$, $\delta(CC_{thr}C_{thr})$, $\delta(OC_{PhN}C_{PhN})$, $\delta(C_{Ph}OC_{PhN})$, $\delta(C_{thr}C_{thr}C_{thr})_{ring}$, waving- $\sigma(C_{Ph}{-}H)$, waving- $\sigma(O{-}C_{Ph})$, waving- $\sigma(C_{thr}{-}H)$, bending- $\rho(C_{PhN}C_{N}N)$, $\tau(C_{Ph}C_{Ph}C_{Ph}C_{Ph})_{ring}$, $\tau(CC_{thr}C_{thr}C_{thr})$, $\tau(C_{thr}C_{thr}C_{thr}C_{thr})_{ring}$
A	650	651	650	651	632.38	$\delta(CCC)_{C4}$, $\delta(CC_{thr}C_{thr})$, $\delta(C_{thr}C_{thr}C_{thr})_{ring}$, $\delta(C_{thr}C_{thr}H)_{ring}$, waving- $\sigma(C_{Ph}{-}H)$, waving- $\sigma(O{-}C_{Ph})$, bending- $\rho(C_{PhN}C_{N}N)$, $\tau(C_{Ph}C_{Ph}C_{Ph}C_{Ph})_{ring}$, $\tau(C_{Ph}CC_{thr}C_{thr})$
A	647	647	647	647		$\delta(C_{Ph}C_{Ph}C_{Ph})_{ring}$, $\delta(C_{Ph}C_{Ph}H)$, $\delta(CCC)_{C4}$, $\delta(CC_{thr}C_{thr})$, $\delta(C_{thr}C_{thr}C_{thr})_{ring}$, waving- $\sigma(C_{thr}{-}H)$, waving- $\sigma(O{-}C_{PhN})$, waving- $\sigma(C_{PhN}{-}C_{N})$, bending- $\rho(C_{PhN}C_{N}N)$, $\tau(C_{Ph}C_{Ph}CC_{thr})$, $\tau(C_{Ph}C_{Ph}OC_{PhN})$, $\tau(C_{Ph}OC_{PhN}C_{PhN})$, $\tau(CC_{thr}C_{thr}C_{thr})$, $\tau(C_{thr}C_{thr}C_{thr}C_{thr})_{ring}$, $\tau(C_{PhN}C_{PhN}C_{PhN}C_{PhN})_{ring}$
A	641	641	643	643		$\delta(C_{Ph}C_{Ph}C_{Ph})_{ring}$, waving- $\sigma(O{-}C_{PhN})$, waving- $\sigma(C_{PhN}{-}C_{N})$, waving- $\sigma(C_{PhN}{-}H)$, bending- $\rho(C_{PhN}C_{N}N)$, $\tau(C_{Ph}C_{Ph}OC_{PhN})$, $\tau(C_{Ph}OC_{PhN}C_{PhN})$, $\tau(C_{PhN}C_{PhN}C_{PhN}C_{PhN})_{ring}$

A	638	640	638	638		$\delta(C_{thr}C_{thr}C_{thr})_{ring}$, $\delta(C_{thr}C_{thr}H)$, $\delta(CCC)_{C4}$
A	636	637	637	637		$\delta(CCC)_{C4}$, $\delta(C_{thr}C_{thr}C_{thr})_{ring}$, $\delta(C_{thr}C_{thr}H)_{ring}$, waving- $\sigma(O\text{-}C_{Ph})$, bending- $\rho(C_{PhN}C_{N}N)$, $\delta(C_{Ph}C_{Ph}C_{Ph})_{ring}$
A	636	636	637	636		$\delta(C_{thr}C_{thr}C_{thr})_{ring}$, $\delta(C_{thr}C_{thr}H)$, $\tau(C_{thr}C_{thr}C_{thr}C_{thr})_{ring}$
A	617	620	618	621		$\delta(C_{Ph}C_{Ph}C_{Ph})_{ring}$, $\delta(C_{Ph}OC_{PhN})$, $\delta(CCC)_{C4}$, $\delta(C_{PhN}C_{PhN}C_{N})_{ring}$, waving- $\sigma(C_{Ph}\text{-}H)$, waving- $\sigma(C\text{-}C_{Ph})$, waving- $\sigma(O\text{-}C_{Ph})$, waving- $\sigma(C_{thr}\text{-}H)$, bending- $\rho(C_{PhN}C_{N}N)$, $\tau(C_{Ph}C_{Ph}C_{Ph}C_{Ph})_{ring}$, $\tau(C_{Ph}C_{Ph}OC_{PhN})$, $\tau(CC_{thr}C_{thr}C_{thr})$, $\tau(C_{thr}C_{thr}C_{thr}C_{thr})_{ring}$
A	576	576	590	586		$\delta(C_{Ph}C_{Ph}C_{Ph})_{ring}$, $\delta(C_{Ph}OC_{PhN})$, $\delta(CCC)_{C4}$, $\delta(C_{PhN}C_{PhN}C_{PhN})_{ring}$, $\delta(C_{PhN}C_{PhN}C_{N})_{ring}$, waving- $\sigma(C_{Ph}\text{-}H)$, waving- $\sigma(C\text{-}C_{Ph})$, waving- $\sigma(O\text{-}C_{Ph})$, waving- $\sigma(C\text{-}C_{thr})$, waving- $\sigma(C_{thr}\text{-}H)$, bending- $\rho(C_{PhN}C_{N}N)$, $\delta(C_{Ph}C_{Ph}C_{Ph})_{ring}$, $\tau(C_{Ph}C_{Ph}CC_{thr})$, $\tau(C_{Ph}C_{Ph}OC_{PhN})$, $\tau(CC_{thr}C_{thr}C_{thr})$, $\tau(C_{thr}C_{thr}C_{thr}C_{thr})_{ring}$
A	549	550	550	553	522.44	waving- $\sigma(C_{PhN}\text{-}C_{N})$, bending- $\rho(C_{PhN}C_{N}N)$, waving- $\sigma(O\text{-}C_{PhN})$, waving- $\sigma(C_{PhN}\text{-}H)$, $\tau(C_{Ph}C_{Ph}OC_{PhN})$, $\tau(C_{Ph}OC_{PhN}C_{PhN})$, $\tau(OC_{PhN}C_{PhN}C_{PhN})$, $\tau(C_{PhN}C_{PhN}C_{PhN}C_{PhN})_{ring}$
A	548	549	548	548		$\delta(CCC)_{C4}$, $\delta(C_{PhN}C_{PhN}C_{PhN})_{ring}$, waving- $\sigma(C\text{-}C_{thr})$, waving- $\sigma(C_{thr}\text{-}H)$, bending- $\rho(C_{PhN}C_{N}N)$, $\delta(C_{Ph}C_{Ph}C_{Ph})_{ring}$, $\tau(C_{Ph}C_{Ph}CC_{thr})$, $\tau(CC_{thr}C_{thr}C_{thr})$, $\tau(C_{thr}C_{thr}C_{thr}C_{thr})_{ring}$
A	536	528	540	530		$\delta(CCC)_{C4}$, $\delta(C_{Ph}OC_{PhN})$, waving- $\sigma(O\text{-}C_{Ph})$, waving- $\sigma(C\text{-}C_{thr})$, waving- $\sigma(C_{thr}\text{-}H)$, $\tau(C_{Ph}C_{Ph}C_{Ph}C_{Ph})_{ring}$, $\tau(C_{Ph}CC_{thr}C_{thr})$, $\tau(CC_{thr}C_{thr}C_{thr})$, $\tau(C_{thr}C_{thr}C_{thr}C_{thr})_{ring}$
A	508	520	511	520		$\delta(C_{PhN}C_{PhN}C_{N})$, waving- $\sigma(C\text{-}C_{Ph})$, waving- $\sigma(O\text{-}C_{Ph})$, waving- $\sigma(C\text{-}C_{thr})$, waving- $\sigma(C_{thr}\text{-}H)$, bending- $\rho(C_{PhN}C_{N}N)$, $\tau(C_{Ph}C_{Ph}C_{Ph}C_{Ph})_{ring}$, $\tau(C_{Ph}C_{Ph}CC_{thr})$, $\tau(C_{Ph}C_{Ph}OC_{PhN})$, $\tau(CC_{thr}C_{thr}C_{thr})$, $\tau(C_{thr}C_{thr}C_{thr}C_{thr})_{ring}$
A	502	503	502	502		waving- $\sigma(C\text{-}C_{Ph})$, waving- $\sigma(O\text{-}C_{Ph})$, waving- $\sigma(C\text{-}C_{thr})$, waving- $\sigma(C_{thr}\text{-}H)$,$\tau(C_{Ph}C_{Ph}C_{Ph}C_{Ph})_{ring}$, $\tau(C_{Ph}C_{Ph}CC_{thr})$, $\tau(C_{Ph}C_{Ph}OC_{PhN})$, $\tau(C_{Ph}CC_{thr}C_{thr})$, $\tau(CC_{thr}C_{thr}C_{thr})$, $\tau(C_{thr}C_{thr}C_{thr}C_{thr})_{ring}$
A	476	473	482	480		$\delta(C_{Ph}OC_{PhN})$, $\delta(OC_{PhN}C_{PhN})$, $\delta(C_{PhN}C_{PhN}C_{PhN})_{ring}$, $\delta(C_{PhN}C_{PhN}H)$, $\delta(C_{PhN}C_{PhN}C_{N})_{ring}$, waving- $\sigma(C_{Ph}\text{-}H)$, waving- $\sigma(C\text{-}C_{Ph})$, waving- $\sigma(O\text{-}C_{Ph})$, waving- $\sigma(C\text{-}C_{thr})$, waving- $\sigma(C_{thr}\text{-}H)$, bending- $\rho(C_{PhN}C_{N}N)$, $\tau(C_{Ph}C_{Ph}C_{Ph}C_{Ph})_{ring}$, $\tau(C_{Ph}C_{Ph}CC_{thr})$, $\tau(C_{Ph}OC_{PhN}C_{PhN})$, $\tau(C_{Ph}CC_{thr}C_{thr})$, $\tau(C_{thr}C_{thr}C_{thr}C_{thr})_{ring}$
A	462	463	462	462		$\delta(OC_{Ph}C_{Ph})$, waving- $\sigma(O\text{-}C_{PhN})$, waving- σ(C-

						H)$_{PhN}$, waving- σ(C-C_{N})$_{PhN}$, bending- ρ($C_{PhN}C_{N}$N), τ($C_{Ph}C_{Ph}OC_{PhN}$), τ($C_{Ph}OC_{PhN}C_{PhN}$), τ($OC_{PhN}C_{PhN}C_{PhN}$), τ($C_{PhN}C_{PhN}C_{PhN}C_{PhN}$)$_{ring}$
A	446	444	437	436		ν(C_{PhN}-C_{N}), δ($C_{Ph}C_{Ph}C_{Ph}$)$_{ring}$, δ($OC_{Ph}C_{Ph}$), δ($C_{Ph}OC_{PhN}$), δ($OC_{PhN}C_{PhN}$), δ($C_{PhN}C_{PhN}C_{PhN}$)$_{ring}$, δ($C_{PhN}C_{PhN}$H)$_{ring}$, δ($C_{PhN}C_{PhN}C_{N}$)$_{ring}$, waving- σ(C-C_{Ph}), waving- σ(O-C_{Ph}), waving- σ(C-C_{thr}), bending- ρ($C_{PhN}C_{N}$N), τ($C_{Ph}C_{Ph}C_{Ph}C_{Ph}$)$_{ring}$, τ($C_{Ph}CC_{thr}C_{thr}$), τ($C_{thr}C_{thr}C_{thr}C_{thr}$)$_{ring}$, τ($C_{PhN}C_{PhN}C_{PhN}C_{PhN}$)$_{ring}$
A	436	436	427	428		waving- σ(C_{Ph}-H)$_{Ph}$, waving- σ(C_{thr}-H), τ($C_{Ph}C_{Ph}C_{Ph}C_{Ph}$)$_{ring}$, τ($C_{Ph}C_{Ph}CC_{thr}$), τ($CC_{thr}C_{thr}C_{thr}$), τ($C_{thr}C_{thr}C_{thr}C_{thr}$)$_{ring}$,
A	426	428	426	424		δ($OC_{Ph}C_{Ph}$), waving- σ(C_{Ph}-H), waving- σ(C_{thr}-H), τ($C_{Ph}C_{Ph}C_{Ph}C_{Ph}$)$_{ring}$, τ($C_{Ph}C_{Ph}CC_{thr}$), τ($C_{Ph}OC_{PhN}C_{PhN}$), τ($CC_{thr}C_{thr}C_{thr}$), τ($C_{thr}C_{thr}C_{thr}C_{thr}$)$_{ring}$, τ($C_{PhN}C_{PhN}C_{PhN}C_{PhN}$)$_{ring}$
A	423	424	421	422		δ($C_{Ph}C_{Ph}C_{Ph}$)$_{ring}$, δ($CC_{Ph}C_{Ph}$), δ($C_{Ph}C_{Ph}$H)$_{ring}$, δ($OC_{Ph}C_{Ph}$), waving- σ(C_{thr}-H), bending- ρ($C_{PhN}C_{N}$N), τ($C_{Ph}C_{Ph}C_{Ph}C_{Ph}$)$_{ring}$, τ($C_{Ph}OC_{PhN}C_{PhN}$), τ($C_{Ph}C_{Ph}OC_{PhN}$), τ($C_{Ph}CC_{thr}C_{thr}$), τ($CC_{thr}C_{thr}C_{thr}$), τ($C_{thr}C_{thr}C_{thr}C_{thr}$)$_{ring}$
A	421	421	420	420		δ($OC_{Ph}C_{Ph}$), waving- σ(C_{thr}-H), τ($C_{Ph}C_{Ph}OC_{PhN}$), τ($C_{Ph}OC_{PhN}C_{PhN}$), τ($C_{Ph}CC_{thr}C_{thr}$), τ($CC_{thr}C_{thr}C_{thr}$), τ($C_{thr}C_{thr}C_{thr}C_{thr}$)$_{ring}$, τ($C_{PhN}C_{PhN}C_{PhN}C_{PhN}$)$_{ring}$
A	418	418	418	417		waving- σ(C_{thr}-H), τ($C_{PhN}C_{PhN}C_{PhN}C_{PhN}$)$_{ring}$, τ($C_{Ph}C_{Ph}OC_{PhN}$), τ($C_{Ph}CC_{thr}C_{thr}$), τ($CC_{thr}C_{thr}C_{thr}$), τ($C_{thr}C_{thr}C_{thr}C_{thr}$)$_{ring}$
A	396	396	395	395		waving- σ(O-C_{PhN}), waving- σ(C_{PhN}-H), waving- σ(C_{PhN}-C_{N}), bending- ρ($C_{PhN}C_{N}$N), τ($C_{Ph}OC_{PhN}C_{PhN}$), τ($OC_{PhN}C_{PhN}C_{PhN}$), τ($C_{PhN}C_{PhN}C_{PhN}C_{PhN}$)$_{ring}$
A	366	363	366	363		δ(CCC)$_{C4}$, δ($OC_{PhN}C_{PhN}$), δ($CC_{thr}C_{thr}$), δ($C_{PhN}C_{PhN}C_{PhN}$)$_{ring}$, waving- σ(C_{Ph}-H), waving- σ(C-C_{Ph}), waving- σ(O-C_{Ph}), waving- σ(C-C_{thr}), waving- σ(C_{thr}-H), bending- ρ($C_{PhN}C_{N}$N), τ($C_{PhN}C_{PhN}C_{PhN}C_{PhN}$)$_{ring}$, τ($C_{Ph}C_{Ph}CC_{thr}$), τ($C_{Ph}C_{Ph}OC_{PhN}$), τ($C_{Ph}CC_{thr}C_{thr}$), τ($C_{thr}C_{thr}C_{thr}C_{thr}$)$_{ring}$
A	347	350	346	348		ν(C_{Ph}-C), δ(CCC)$_{C4}$, δ($OC_{Ph}C_{Ph}$), δ($C_{Ph}OC_{PhN}$), δ($OC_{PhN}C_{PhN}$), δ($CC_{thr}C_{thr}$), δ($C_{PhN}C_{PhN}C_{PhN}$)$_{ring}$, δ($C_{PhN}C_{PhN}$H), δ($C_{PhN}C_{PhN}C_{N}$)$_{ring}$, waving- σ(O-C_{Ph}), waving- σ(C-C_{thr}), τ($C_{Ph}C_{Ph}C_{Ph}C_{Ph}$)$_{ring}$, τ($C_{Ph}OC_{PhN}C_{PhN}$), τ($C_{thr}C_{thr}C_{thr}C_{thr}$)$_{ring}$
A	324	325	321	324		ν(C_{thr}-C), δ($OC_{Ph}C_{Ph}$), δ(CCC)$_{C4}$, δ($OC_{PhN}C_{PhN}$), δ($CC_{thr}C_{thr}$), δ($C_{thr}C_{thr}C_{thr}$)$_{ring}$, bending- ρ($C_{PhN}C_{N}$N), τ($C_{Ph}C_{Ph}OC_{PhN}$), τ($C_{Ph}OC_{PhN}C_{PhN}$),

						$\tau(CC_{thr}C_{thr}C_{thr})$, $\tau(C_{thr}C_{thr}C_{thr}C_{thr})_{ring}$, $\tau(OC_{PhN}C_{PhN}C_{PhN})$
A	295	294	298	293		$\delta(CC_{Ph}C_{Ph})$, $\delta(CCC)_{C4}$, $\delta(CC_{thr}C_{thr})$, waving- $\sigma(C\text{-}C_{Ph})$, waving- $\sigma(C\text{-}C_{thr})$, waving- $\sigma(C_{thr}\text{-}H)$, $\tau(C_{Ph}C_{Ph}OC_{PhN})$, $\tau(C_{Ph}OC_{PhN}C_{PhN})$, $\tau(C_{Ph}CC_{thr}C_{thr})$, $\tau(CC_{thr}C_{thr}C_{thr})$, $\tau(C_{thr}C_{thr}C_{thr}C_{thr})_{ring}$, $\tau(OC_{PhN}C_{PhN}C_{PhN})$
A	287	287	286	287		$\nu(C_{thr}\text{-}C)$, $\delta(CC_{Ph}C_{Ph})$, $\delta(CCC)_{C4}$, $\delta(CC_{thr}C_{thr})$, $\delta(C_{thr}C_{thr}C_{thr})_{ring}$, waving- $\sigma(C_{thr}\text{-}H)$, waving- $\sigma(C_{PhN}\text{ -}C_{N})$, bending- $\rho(C_{PhN}C_{N}N)$, $\tau(C_{Ph}C_{Ph}OC_{PhN})$, $\tau(C_{Ph}OC_{PhN}C_{PhN})$, $\tau(CC_{thr}C_{thr}C_{thr})$, $\tau(C_{thr}C_{thr}C_{thr}C_{thr})_{ring}$, $\tau(OC_{PhN}C_{PhN}C_{PhN})$
A	285	285	285	285		$\nu(C_{thr}\text{-}C)$, $\delta(CC_{Ph}C_{Ph})$, $\delta(CCC)_{C4}$, $\delta(CC_{thr}C_{thr})$, $\delta(C_{thr}C_{thr}C_{thr})_{ring}$, waving- $\sigma(C\text{-}C_{Ph})$, waving- $\sigma(C_{thr}\text{-}H)$, bending- $\rho(C_{PhN}C_{N}N)$, $\tau(C_{Ph}C_{Ph}CC_{thr})$, $\tau(C_{Ph}C_{Ph}OC_{PhN})$, $\tau(C_{Ph}OC_{PhN}C_{PhN})$, $\tau(CC_{thr}C_{thr}C_{thr})$, $\tau(C_{thr}C_{thr}C_{thr}C_{thr})_{ring}$, $\tau(OC_{PhN}C_{PhN}C_{PhN})$
A	267	266	268	265		$\nu(C_{thr}\text{-}C)$, $\delta(CCC)_{C4}$, $\delta(CC_{thr}C_{thr})$, $\delta(C_{thr}C_{thr}C_{thr})_{ring}$, waving- $\sigma(C\text{-}C_{Ph})$, waving- $\sigma(C_{thr}\text{-}H)$, $\tau(C_{Ph}C_{Ph}C_{Ph}C_{Ph})_{ring}$, $\tau(C_{Ph}C_{Ph}CC_{thr})$, $\tau(CC_{thr}C_{thr}C_{thr})$, $\tau(C_{thr}C_{thr}C_{thr}C_{thr})_{ring}$
A	258	261	257	259		$\delta(CC_{Ph}C_{Ph})$, $\delta(CC_{thr}C_{thr})$, waving- $\sigma(C_{thr}\text{-}H)$, waving- $\sigma(O\text{-}C_{PhN})$, waving- $\sigma(C_{PhN}\text{-}H)$, waving- $\sigma(C_{PhN}\text{-}C_{N})$, bending- $\rho(C_{PhN}C_{N}N)$, $\tau(C_{Ph}C_{Ph}C_{Ph}C_{Ph})_{ring}$, $\tau(C_{Ph}C_{Ph}OC_{PhN})$, $\tau(C_{Ph}OC_{PhN}C_{PhN})$, $\tau(CC_{thr}C_{thr}C_{thr})$, $\tau(C_{thr}C_{thr}C_{thr}C_{thr})_{ring}$, $\tau(OC_{PhN}C_{PhN}C_{PhN})$, $\tau(C_{PhN}C_{PhN}C_{PhN}C_{PhN})_{ring}$
A	246	248	246	248		$\delta(CC_{thr}C_{thr})$, waving- $\sigma(C_{thr}\text{-}H)$, waving- $\sigma(O\text{-}C_{PhN})$, waving- $\sigma(C_{PhN}\text{-}H)$, bending- $\rho(C_{PhN}C_{N}N)$, $\tau(C_{Ph}C_{Ph}CC_{thr})$, $\tau(C_{Ph}C_{Ph}OC_{PhN})$, $\tau(C_{Ph}OC_{PhN}C_{PhN})$, $\tau(C_{Ph}CC_{thr}C_{thr})$, $\tau(CC_{thr}C_{thr}C_{thr})$, $\tau(C_{thr}C_{thr}C_{thr}C_{thr})_{ring}$, $\tau(OC_{PhN}C_{PhN}C_{PhN})$, $\tau(C_{PhN}C_{PhN}C_{PhN}C_{PhN})_{ring}$
A	236	230	236	229		$\delta(C_{Ph}OC_{PhN})$, $\delta(OC_{PhN}C_{PhN})$, $\delta(CC_{thr}C_{thr})$, $\delta(CCC_{N})$, waving- $\sigma(O\text{-}C_{Ph})$, waving- $\sigma(C_{thr}\text{-}H)$, bending- $\rho(C_{PhN}C_{N}N)$, $\tau(C_{Ph}C_{Ph}C_{Ph}C_{Ph})_{ring}$, $\tau(C_{Ph}C_{Ph}CC_{thr})$, $\tau(C_{Ph}C_{Ph}OC_{PhN})$, $\tau(C_{Ph}CC_{thr}C_{thr})$, $\tau(CC_{thr}C_{thr}C_{thr})$, $\tau(C_{thr}C_{thr}C_{thr}C_{thr})_{ring}$
A	222	221	223	221		$\delta(CC_{Ph}C_{Ph})$, $\delta(OC_{Ph}C_{Ph})$, $\delta(CC_{thr}C_{thr})$, waving- $\sigma(C\text{-}C_{thr})$, waving- $\sigma(C_{thr}\text{-}H)$, waving- $\sigma(C_{PhN}\text{-}C_{N})$, bending- $\rho(C_{PhN}C_{N}N)$, $\tau(C_{Ph}C_{Ph}CC_{thr})$, $\tau(C_{Ph}C_{Ph}OC_{PhN})$, $\tau(C_{Ph}OC_{PhN}C_{PhN})$, $\tau(C_{Ph}CC_{thr}C_{thr})$, $\tau(CC_{thr}C_{thr}C_{thr})$, $\tau(C_{thr}C_{thr}C_{thr}C_{thr})_{ring}$, $\tau(OC_{PhN}C_{PhN}C_{PhN})$, $\tau(C_{PhN}C_{PhN}C_{PhN}C_{PhN})_{ring}$
A	184	196	183	193		$\nu(C_{Ph}\text{-}C)$, $\delta(C_{Ph}C_{Ph}C_{Ph})_{ring}$, $\delta(CCC)_{C4}$,

						δ($C_{Ph}OC_{PhN}$), δ($CC_{thr}C_{thr}$), δ($C_{PhN}C_{PhN}C_{PhN}$)$_{ring}$, waving- σ(C_{Ph}-H), bending- ρ($C_{PhN}C_{N}N$), τ($C_{Ph}C_{Ph}C_{Ph}C_{Ph}$)$_{ring}$, τ($C_{Ph}CC_{thr}C_{thr}$), τ($CC_{thr}C_{thr}C_{thr}$)
A	152	147	148	144		δ(CCC)$_{C4}$, δ($C_{PhN}C_{PhN}C_{N}$), waving- σ(C_{Ph}-H), waving- σ(C-C_{Ph}), waving- σ(O-C_{Ph}), bending- ρ($C_{PhN}C_{N}N$), τ($C_{Ph}C_{Ph}C_{Ph}C_{Ph}$)$_{ring}$, τ($C_{Ph}CC_{thr}C_{thr}$), τ($C_{Ph}C_{Ph}OC_{PhN}$), τ($CC_{thr}C_{thr}C_{thr}$)
A	142	143	144	142		waving- σ(O-C_{PhN}), waving- σ(C_{PhN}-H), waving- σ(C_{PhN}-C_{N}), bending- ρ($C_{PhN}C_{N}N$), τ($C_{Ph}C_{Ph}CC_{thr}$), τ($C_{Ph}C_{Ph}OC_{PhN}$), τ($C_{Ph}OC_{PhN}C_{PhN}$), τ($C_{Ph}CC_{thr}C_{thr}$), τ($CC_{thr}C_{thr}C_{thr}$), τ($OC_{PhN}C_{PhN}C_{PhN}$), τ($C_{PhN}C_{PhN}C_{PhN}C_{PhN}$)$_{ring}$
A	118	118	115	116		δ($C_{PhN}C_{PhN}C_{PhN}$)$_{ring}$, δ($C_{PhN}C_{PhN}C_{N}$), δ($C_{PhN}C_{PhN}H$), waving- σ(C-C_{Ph}), waving- σ(O-C_{Ph}), bending- ρ($C_{PhN}C_{N}N$), τ($C_{Ph}C_{Ph}OC_{PhN}$),
A	110	110	107	110		δ(CCC)$_{C4}$, waving- σ(C_{PhN}-C_{N}), τ($C_{Ph}C_{Ph}C_{Ph}C_{Ph}$)$_{ring}$, τ($C_{Ph}C_{Ph}OC_{PhN}$), τ($C_{Ph}CC_{thr}C_{thr}$), τ($CC_{thr}C_{thr}C_{thr}$), τ($C_{thr}C_{thr}C_{thr}C_{thr}$)$_{ring}$, τ($C_{PhN}C_{PhN}C_{PhN}C_{PhN}$)$_{ring}$
A	99	100	97	98		δ(CCC)$_{C4}$, waving- σ(C-C_{thr}), waving- σ(C_{thr}-H), waving- σ(C_{PhN}-H), waving- σ(C_{PhN}-C_{N}), bending- ρ($C_{PhN}C_{N}N$), τ($C_{Ph}CC_{thr}C_{thr}$), τ($C_{Ph}C_{Ph}OC_{PhN}$), τ($C_{Ph}OC_{PhN}C_{PhN}$), τ($C_{Ph}CC_{thr}C_{thr}$), τ($CC_{thr}C_{thr}C_{thr}$), τ($C_{thr}C_{thr}C_{thr}C_{thr}$)$_{ring}$, τ($OC_{PhN}C_{PhN}C_{PhN}$), τ($C_{PhN}C_{PhN}C_{PhN}C_{PhN}$)$_{ring}$
A	91	89	91	86		δ(CCC)$_{C4}$, waving- σ(C-C_{thr}), waving- σ(C_{thr}-H), waving- σ(C_{PhN}-C_{N}), bending- ρ($C_{PhN}C_{N}N$), τ($C_{Ph}C_{Ph}C_{Ph}C_{Ph}$)$_{ring}$, τ($C_{Ph}C_{Ph}OC_{PhN}$), τ($C_{Ph}OC_{PhN}C_{PhN}$), τ($C_{Ph}CC_{thr}C_{thr}$), τ($CC_{thr}C_{thr}C_{thr}$), τ($C_{thr}C_{thr}C_{thr}C_{thr}$)$_{ring}$, τ($OC_{PhN}C_{PhN}C_{PhN}$), τ($C_{PhN}C_{PhN}C_{PhN}C_{PhN}$)$_{ring}$
A	86	87	86	85		δ($OC_{Ph}C_{Ph}$), δ(CCC)$_{C4}$, waving- σ(C_{PhN}-H), waving- σ(C_{PhN}-C_{N}), bending- ρ($C_{PhN}C_{N}N$), τ($C_{Ph}C_{Ph}C_{Ph}C_{Ph}$)$_{ring}$, τ($C_{Ph}C_{Ph}CC_{thr}$), τ($C_{Ph}C_{Ph}OC_{PhN}$), τ($C_{Ph}OC_{PhN}C_{PhN}$), τ($C_{Ph}CC_{thr}C_{thr}$), τ($CC_{thr}C_{thr}C_{thr}$), τ($C_{thr}C_{thr}C_{thr}C_{thr}$)$_{ring}$, τ($OC_{PhN}C_{PhN}C_{PhN}$), τ($C_{PhN}C_{PhN}C_{PhN}C_{PhN}$)$_{ring}$
A	74	75	76	73		δ(CCC)$_{C4}$, δ($CC_{thr}C_{thr}$), waving- σ(C-C_{thr}), waving- σ(C_{Ph}-H), waving- σ(C_{thr}-H), waving- σ(C_{PhN}-C_{N}), bending- ρ($C_{PhN}C_{N}N$), τ($C_{Ph}C_{Ph}CC_{thr}$), τ($C_{Ph}CC_{thr}C_{thr}$), τ($CC_{thr}C_{thr}C_{thr}$), τ($C_{thr}C_{thr}C_{thr}C_{thr}$)$_{ring}$, τ($OC_{PhN}C_{PhN}C_{PhN}$), τ($C_{PhN}C_{PhN}C_{PhN}C_{PhN}$)$_{ring}$
A	62	64	64	65		δ($CC_{Ph}C_{Ph}$), δ(CCC)$_{C4}$, waving- σ(C-C_{Ph}), waving- σ(C-C_{thr}), waving- σ(C_{thr}-H), waving-

						$\sigma(C_{PhN}\text{-}C_{N})$, bending- $\rho(C_{PhN}C_{N}N)$, $\tau(C_{Ph}C_{Ph}C_{Ph}C_{Ph})_{ring}$, $\tau(C_{Ph}C_{Ph}CC_{thr})$, $\tau(C_{Ph}OC_{PhN}C_{PhN})$, $\tau(C_{Ph}CC_{thr}C_{thr})$, $\tau(CC_{thr}C_{thr}C_{thr})$, $\tau(C_{thr}C_{thr}C_{thr}C_{thr})_{ring}$, $\tau(OC_{PhN}C_{PhN}C_{PhN})$, $\tau(C_{PhN}C_{PhN}C_{PhN}C_{PhN})_{ring}$
A	60	62	60	60		$\delta(C_{Ph}OC_{PhN})$, $\delta(CCC)_{C4}$, waving- $\sigma(C\text{-}C_{Ph})$, waving- $\sigma(O\text{-}C_{Ph})$, waving- $\sigma(C\text{-}C_{thr})$, waving- $\sigma(C_{thr}\text{-}H)$, $\tau(C_{Ph}C_{Ph}C_{Ph}C_{Ph})_{ring}$, $\tau(C_{Ph}C_{Ph}OC_{PhN})$, $\tau(C_{Ph}CC_{thr}C_{thr})$, $\tau(CC_{thr}C_{thr}C_{thr})$, $\tau(C_{thr}C_{thr}C_{thr}C_{thr})_{ring}$
A	56	60	57	57		$\delta(CCC)_{C4}$, $\delta(CC_{thr}C_{thr})$, waving- $\sigma(C_{Ph}\text{-}H)$, waving- $\sigma(C\text{-}C_{Ph})$, waving- $\sigma(O\text{-}C_{Ph})$, waving- $\sigma(C\text{-}C_{thr})$, waving- $\sigma(C_{thr}\text{-}H)$, $\tau(C_{Ph}C_{Ph}C_{Ph}C_{Ph})_{ring}$, $\tau(C_{Ph}C_{Ph}CC_{thr})$, $\tau(C_{Ph}C_{Ph}OC_{PhN})$, $\tau(C_{Ph}OC_{PhN}C_{PhN})$, $\tau(C_{Ph}CC_{thr}C_{thr})$, $\tau(CC_{thr}C_{thr}C_{thr})$, $\tau(C_{thr}C_{thr}C_{thr}C_{thr})_{ring}$, $\tau(OC_{PhN}C_{PhN}C_{PhN})$, $\tau(C_{PhN}C_{PhN}C_{PhN}C_{PhN})_{ring}$
A	46	49	48	50		$\delta(CC_{Ph}C_{Ph})$, $\delta(CCC)_{C4}$, $\delta(C_{Ph}OC_{PhN})$, waving- $\sigma(C\text{-}C_{Ph})$, waving- $\sigma(O\text{-}C_{Ph})$, waving- $\sigma(C\text{-}C_{thr})$, waving- $\sigma(C_{thr}\text{-}H)$, bending- $\rho(C_{PhN}C_{N}N)$, $\tau(C_{Ph}C_{Ph}CC_{thr})$, $\tau(C_{Ph}OC_{PhN}C_{PhN})$, $\tau(C_{Ph}CC_{thr}C_{thr})$, $\tau(CC_{thr}C_{thr}C_{thr})$,
A	27	25	27	24		$\delta(CC_{Ph}C_{Ph})$, $\delta(CCC)_{C4}$, waving- $\sigma(C\text{-}C_{thr})$, $\tau(C_{Ph}C_{Ph}C_{Ph}C_{Ph})_{ring}$, $\tau(C_{Ph}C_{Ph}CC_{thr})$, $\tau(C_{Ph}C_{Ph}OC_{PhN})$, $\tau(C_{Ph}OC_{PhN}C_{PhN})$, $\tau(C_{Ph}CC_{thr}C_{thr})$, $\tau(CC_{thr}C_{thr}C_{thr})$, $\tau(C_{PhN}C_{PhN}C_{PhN}C_{PhN})_{ring}$
A	20	21	20	20		$\delta(C_{Ph}OC_{PhN})$, $\delta(CCC)_{C4}$, waving- $\sigma(C_{Ph}\text{-}H)$, waving- $\sigma(C\text{-}C_{Ph})$, waving- $\sigma(C\text{-}C_{thr})$, waving- $\sigma(O\text{-}C_{PhN})$, $\tau(C_{Ph}C_{Ph}C_{Ph}C_{Ph})_{ring}$, $\tau(C_{Ph}C_{Ph}CC_{thr})$, $\tau(C_{Ph}C_{Ph}OC_{PhN})$, $\tau(C_{Ph}OC_{PhN}C_{PhN})$, $\tau(C_{Ph}CC_{thr}C_{thr})$, $\tau(CC_{thr}C_{thr}C_{thr})$
A	15	15	19	16		$\delta(CCC)_{C4}$, waving- $\sigma(C\text{-}C_{Ph})$, waving- $\sigma(O\text{-}C_{PhN})$, waving- $\sigma(C_{Ph}\text{-}H)$, waving- $\sigma(C\text{-}C_{thr})$, $\tau(C_{Ph}C_{Ph}C_{Ph}C_{Ph})_{ring}$, $\tau(C_{Ph}C_{Ph}CC_{thr})$, $\tau(C_{Ph}C_{Ph}OC_{PhN})$, $\tau(C_{Ph}OC_{PhN}C_{PhN})$, $\tau(C_{Ph}CC_{thr}C_{thr})$, $\tau(CC_{thr}C_{thr}C_{thr})$
A	5	8	8	4		$\delta(OC_{Ph}C_{Ph})$, $\delta(CCC)_{C4}$, waving- $\sigma(C\text{-}C_{Ph})$, waving- $\sigma(C\text{-}C_{thr})$, waving- $\sigma(O\text{-}C_{Ph})$, $\tau(C_{Ph}C_{Ph}CC_{thr})$, $\tau(C_{Ph}C_{Ph}OC_{PhN})$, $\tau(C_{Ph}OC_{PhN}C_{PhN})$, $\tau(C_{Ph}CC_{thr}C_{thr})$, $\tau(CC_{thr}C_{thr}C_{thr})$, $\tau(OC_{PhN}C_{PhN}C_{PhN})$

[1]for *cis*-conformer II

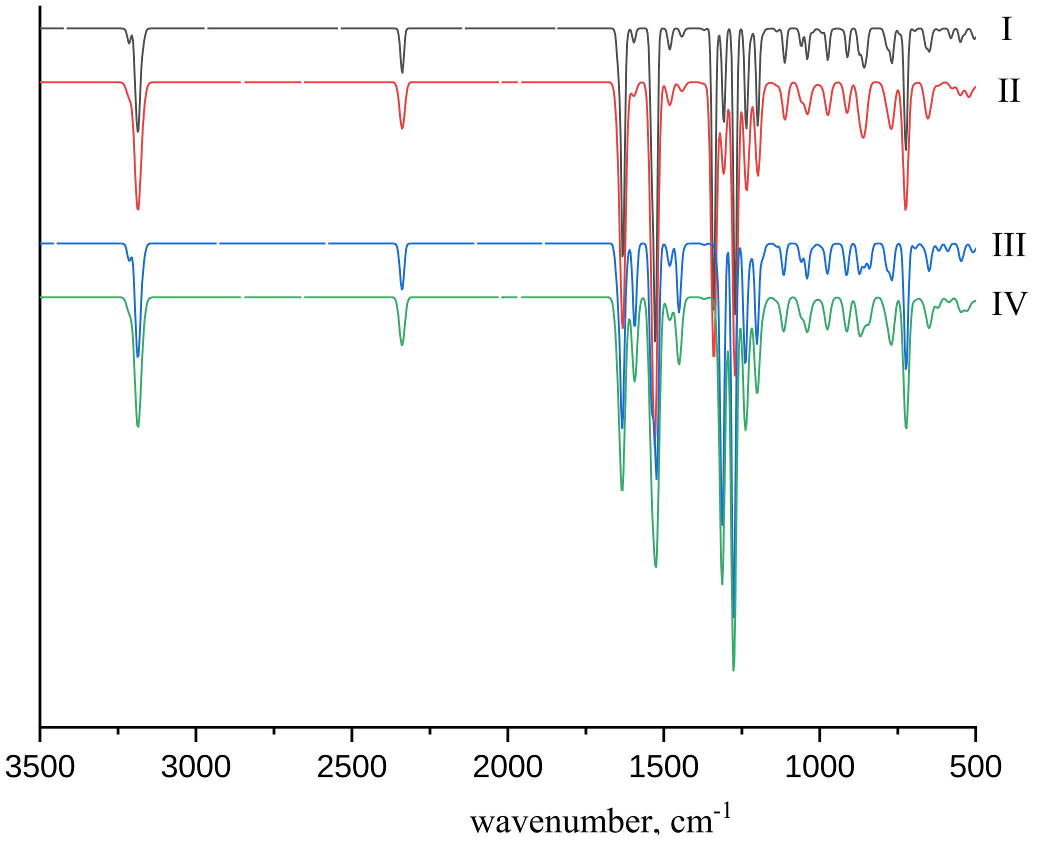

Figure S1. The calculated IR spectra of the conformers I and II (*cis*) and III and IV (*trans*)

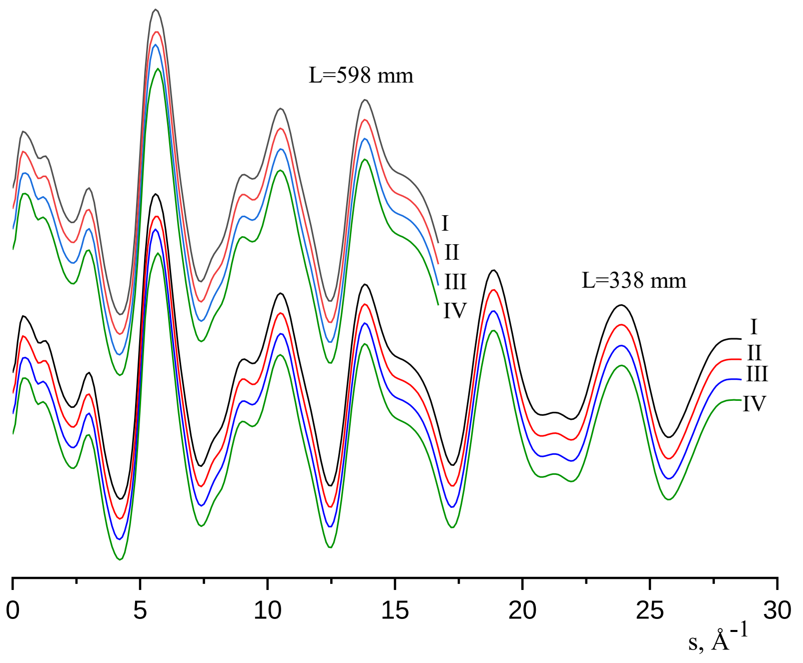

Figure S2. Theoretical molecular intensities sM(s) of conformers I-IV of 4-(4-tritylphenoxy)phthalonitrile.

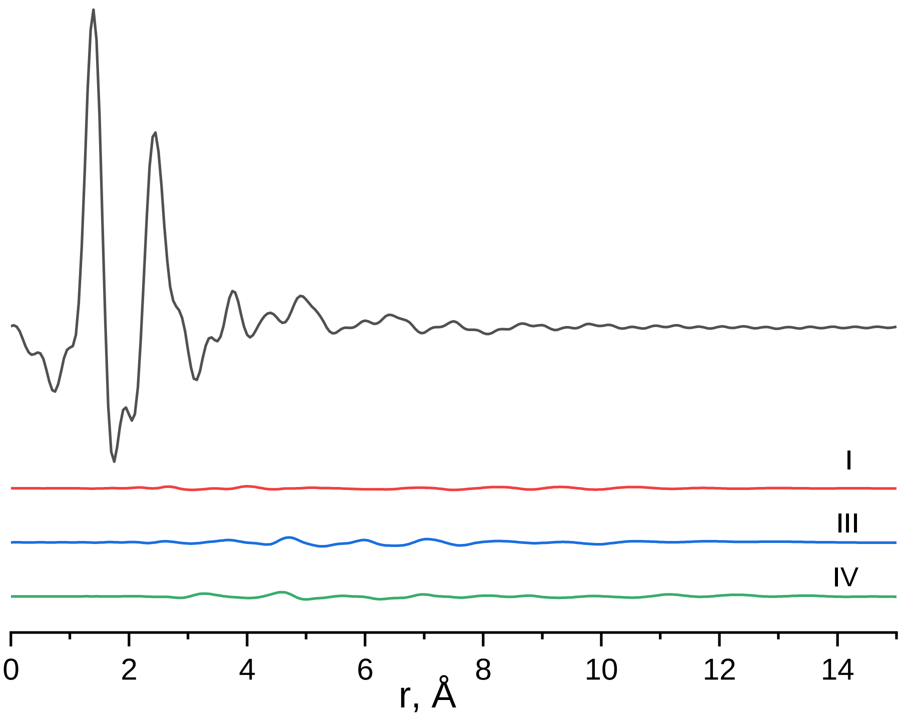

Figure S3. Theoretical radial distribution function f(r) for conformer II and difference Δf(r) for conformers I, III IV.

Table S4. Conditions of the synchronous GED/MS experiment.

Nozzle-to plate distances, mm	338	598
Number of recorded plates	5	6
Electron beam current, μA	1.6	1.2
Wavelength of electrons, Å	0.04020(3)	0.04009(3)
Temperature of the effusion cell, K	475(10)	481(10)
Average exposure time, s	130	70
Residual gas pressure in diffraction chamber, Torr	$1.0\cdot10^{-6}$	$1.5\cdot10^{-6}$
s-range/Δs, $Å^{-1}$	3.0-28.6/0.1	1.3-16.7/0.1
Ionization voltage, V	50	50
